# Soft X-ray Spectroscopy Simulations with Multiconfigurational Wave Function Theory: Spectrum Completeness, Sub-eV Accuracy, and Quantitative Reproduction of Line Shapes

**DOI:** 10.1021/acs.jctc.1c00566

**Published:** 2022-01-24

**Authors:** Francesco Montorsi, Francesco Segatta, Artur Nenov, Shaul Mukamel, Marco Garavelli

**Affiliations:** † Department of Industrial Chemistry “Toso Montanari”, University of Bologna, Viale del Risorgimento, 4, 40136 Bologna, Italy; ‡ Department of Chemistry and Department of Physics & Astronomy, University of California, Irvine, California 92697-2025, United States

## Abstract

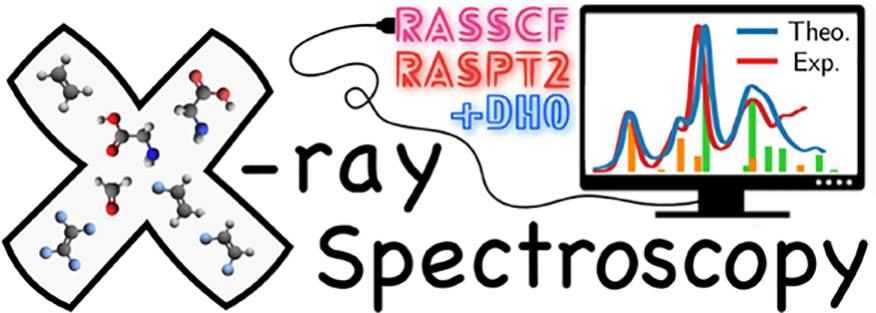

Multireference methods are known for their ability to accurately treat states of very different nature in many molecular systems, facilitating high-quality simulations of a large variety of spectroscopic techniques. Here, we couple the multiconfigurational restricted active space self-consistent field RASSCF/RASPT2 method (of the CASSCF/CASPT2 methods family) to the displaced harmonic oscillator (DHO) model, to simulate soft X-ray spectroscopy. We applied such an RASSCF/RASPT2+DHO approach at the K-edges of various second-row elements for a set of small organic molecules that have been recently investigated at other levels of theory. X-ray absorption near-edge structure (XANES) and X-ray photoelectron spectroscopy (XPS) are simulated with a sub-eV accuracy and a correct description of the spectral line shapes. The method is extremely sensitive to the observed spectral shifts on a series of differently fluorinated ethylene systems, provides spectral fingerprints to distinguish between stable conformers of the glycine molecule, and accurately captures the vibrationally resolved carbon K-edge spectrum of formaldehyde. Differences with other theoretical methods are demonstrated, which show the advantages of employing a multireference/multiconfigurational approach. A protocol to systematically increase the number of core-excited states considered while maintaining a contained computational cost is presented. Insight is eventually provided for the effects caused by removing core–electrons from a given atom in terms of bond rearrangement and influence on the resulting spectral shapes within a unitary orbital-based framework for both XPS and XANES spectra.

## Introduction

Recent developments of synchrotron light sources and X-ray free-electron lasers (XFEL) have opened up a new era of electronic spectroscopy.^1−3^ These offer novel tools for looking at molecular structure and dynamics with unprecedented temporal and spatial resolutions and radiation intensities,^4^ enabling an in-depth investigation of static and dynamical properties of matter, inaccessible in the optical regime. The capacity of X-ray techniques ranges from probing electronic coherences on the attosecond (as) to femtosecond (fs) time scales^5^ to revealing chemical reactions in real time,^6^ spin-crossover dynamics,^7^ and change of electronic^8^ and/or molecular structure in the excited state.^9^

Given the present and upcoming developments, the availability of accurate, robust, and flexible theoretical methods, able to describe the physical phenomena involved in the interaction between X-rays and molecules, is timely and of upmost importance. The increasing complexity of current experiments often gives rise to equally complex spectral signatures, whose direct interpretation is a challenging task: it is therefore necessary to support experimental measurement with adequate theoretical tools. Moreover, theoretical modeling can bring new ideas to the field, guide the development of new experimental techniques, and increase the efficiency of existing ones.

Recent years have witnessed a surge in adapting state-of-the-art quantum mechanical methodologies to target core excitations.^10^ Single reference methods, such as TDDFT, ADC2, and CC-EOM formulations within the core–valence separation scheme (CVS),^11,12^ perform very well for single core excitations from the ground state (typically probed in XANES spectra). Modifications such as the transition potential (TP),^13^ maximum overlap method (MOM),^6^ and *hole–hole* Tamm-Dancoff approximated (hh-TDA)^14^ formulations of DFT allow an extension of the applicability, albeit with some limitations, to multiple excitations. This enables the description of core excitations from valence-excited states, as required by some X-ray techniques such as time-resolved (TR-)XANES.^15^ However, these approaches often face difficulties in predicting absolute state energies and relative transition intensities, requiring ad hoc energy shifts to align the simulations with experiment.^16−18^

Among the ample body of approaches, multireference methods have been also extensively employed, from early MCSCF method developments^19−21^ to more recent applications. Multiconfiguration wave function-based methods such as the restricted active space self-consistent field (RASSCF) approach often coupled to second order perturbation theory (RASPT2) corrections are in fact able by definition of treating states with singly and multiply excited character at equal footing. The RASSCF/RASPT2 approach has been applied to model K- and L-edges of small organic molecules^22,23^ molecular ions^24,25^ as well as transition metal complexes L-edges.^26−29^ Very recently, a formulation of the RASSCF/RASPT2 protocol in the CVS scheme has been implemented,^30^ which enables the selective computation of valence, single-, multiple-core, and mixed core–valence excitations, as well as single and multiple core-ionized states. This protocol has been applied to facilitate TR-XANES simulations^31−33^ and to handle multiple core excitations required for nonlinear techniques, such as 2D coherent X-ray, double quantum coherence, and two-photon absorption.^23,34^

Core-level transitions are characterized by an extremely short lifetime, which translates usually in broad unstructured peaks in the measured spectra. It is extremely common to find spectral shapes described with simple Lorentzian/Gaussian broadening of the computed transitions, whose width is typically fit to match the experiments. Nonetheless, the coupling of the electronic states with nuclear degrees of freedom can also affect the line shape, in terms of additional (asymmetric) broadening and/or appearance of vibronic fine structure. So far, the vibronic effects have been addressed by means of approaches based on potential energy surfaces (PES) mapping, typically complemented by time-dependent wave packet propagation, on small molecular systems and/or along a very restricted number of modes.^24,25,35−37^ This allows an accurate description of the vibrational structure (especially when sensitive techniques such as resonant inelastic X-ray scattering are simulated) and of possible dissociative coordinates but cannot be extended to systems of medium/large size. Evaluation of Franck–Condon overlaps within the harmonic approximation of the PES topology, also known as the DHO (or linear vibronic coupling) model, has been also proposed as a viable approximation^38−41^ but not coupled to RASSCF/RASPT2 quantum chemistry data in this context, to the best of our knowledge.

In this work, we demonstrate the effectiveness of the RASSCF/RASPT2+DHO protocol to simulate first-principles K-edge XANES and XPS spectra for biologically relevant second-row atoms C, N, O, and F with near quantitative accuracy, only relying on quantum chemical quantities computed at the ground state minimum. In particular, we study a set of fluorine substituted ethanes/ethylenes, the glycine molecule in the two highest populated gas phase conformations, as well as the C K-edge spectrum of formaldehyde. A comparison between the RASSCF/RASPT2+DHO approach and results from other levels of theory on the same molecular systems^16,39,42,43^ is systematically carried on.

Comparison to (high resolution) experimental spectra evidence the sub-eV accuracy with which the protocol is able to predict transitions, as well as its ability to resolve shake-up features (i.e., mixed core–valence excitations), to which other methods are blind. The coupling of the electronic and nuclear degrees of freedom within the approximation of the DHO model allows the qualitative reproduction of the vibronic effects (energy shifts, band shapes, fine structure) of the XANES and XPS spectra. Finally, we show how the line shapes can be improved toward a quantitative agreement with experiments by taking into account the different potential energy surface profiles of core excited states with respect to the ground state (Duschinksy effect) and the coordinate dependence of the transition dipole moment (Herzberg–Teller effect). The importance of these effects is rationalized in terms of electron and nuclear relaxation upon vacating core orbitals, which also allowed us to draw an orbital-based qualitative criterion to describe the structural changes in a unique framework for both core-excitation (i.e., in XANES) and core-ionization (i.e., in XPS) processes.

The present approach can simulate spectroscopy from first principles without any feedback from experiment, with a sub-eV absolute error which makes it the protocol of choice for predicting spectral signatures and guiding experiments. It can treat both single- and multiple-core hole states, which make it ideally suitable for linear and nonlinear X-ray spectroscopy. The accuracy of the method puts it in the position to serve as a benchmark for existing and future theoretical methods. The need to actively define the active space composition, often cited as a drawback compared to “black box” methods, allows the capture of the main pre-edge signals at a greatly reduced computational cost by considering a few suitably selected orbitals. An algorithm to systematically increase the number of core-level transitions while keeping the cost of the computations low is also presented, demonstrating a route to make the RASSCF/RASPT2+DHO approach applicable also to medium and large size molecules.

## Theoretical Methodology

The computation of quantum mechanical properties of core excited states in molecular systems poses several challenges: excitation of core-excited/core-ionized states requires light with hundreds to thousands of eV; these states are preceded by a vast number of energetically lower lying valence states. A strategy to specifically target core excitations without the need to evaluate all the lower states is called for. The restricted active space self-consistent field (RASSCF)^44^ approach from the family of active-space/wave function based multiconfigurational methods offers a neat way to tackle this problem. The basic steps of this strategy are virtually identical to those of a standard computation and comprise the following: (a) the selection of the active space (AS), i.e., the set of orbitals and electrons of paramount importance for describing the configuration of the electronic states of interest, and (b) the optimization of the wave function in terms of both orbital shapes and CI coefficients. The active space should comprise the core orbital(s) of interest. On top of that, a novel projection technique named HEXS (highly excited states) allows to set to zero the CI coefficients of all configuration state functions (CFSs) with the maximum occupation from a given subspace, thus effectively projecting them out from the wave function: in this way, by inserting the core orbital(s) of interest in the orbital subspace(s) to which HEXS is applied, single and multiple core-hole configurations (from one or several atom centers) can be directly accessed. This technique further allows modeling of transition dipole moments between valence, singly excited, and doubly excited core states.^22,23,30^ Quantitative agreement with experiment can be achieved by means of multireference second order perturbation correction (RASPT2) on the top of the RASSCF wave function, recovering dynamical correlation for the electrons outside of the active space. The scheme, similarly applied elsewhere,^22,23,30,32,33,45,46^ is implemented in the quantum chemistry packages such as OpenMolcas^47,48^ and Molpro.^49^ The former software has been used for all quantum chemistry calculations reported here.

**Figure 1:**
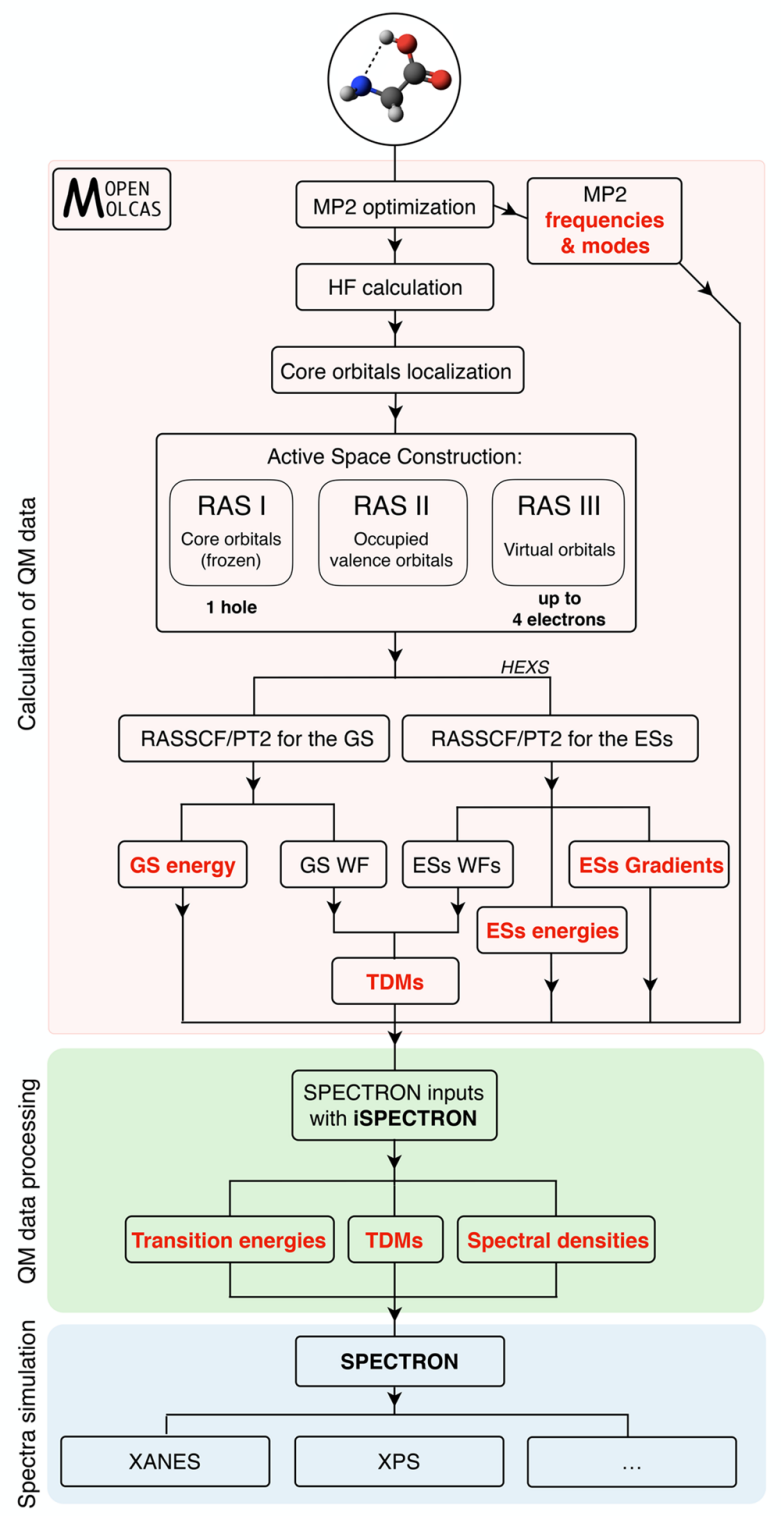
Flowchart of the RASSCF/RASPT2+DHO protocol used for the computation of spectroscopy through SPECTRON. The scheme reports the steps that lead to XANES spectra. Similarly, XPS spectra can be produced when energies and gradients are computed for the cationic states instead of core-excited states, together with Dyson intensities (instead of TDMs). Three main steps are highlighted: the calculation of QM data, their processing, and the simulation of spectroscopy. The possible computation of higher-lying multiple-core excitations with the help of the HEXS projection scheme facilitates nonlinear spectroscopy within the same protocol.

The simulation protocols for XANES and XPS signals are similar. The former, employed here to calculate the spectra of organic molecules at second-row elements K-edges, is reported in Figure 1. The key required ab initio quantum mechanical quantities are state energies (for the ground state (GS) and the core excited state (ES)), transition dipole moments, ground state vibrational frequencies and normal modes, and excited state gradients. The broadening of the simulated signals also accounts for the excited state lifetime, spanning a range of values between 1 and 4 fs (see Section S3 of the SI), reflecting the typical values of pre-edge transitions of the different studied elements.^10^ GS frequencies, normal modes, and excited state energy gradients were utilized to account for the vibronic coupling within the DHO model, which assumes the potential energy surfaces of the electronic states to be identical harmonic wells with (at most) different equilibrium positions.^50^ Spectra were simulated via the SPECTRON software,^51^ a unified platform for optical spectroscopy calculations. This software has been recently interfaced with OpenMolcas thus allowing the semiautomatized generation, plotting, and analysis of the spectra.^50^

A similar strategy has been applied to the computation of XPS spectra and core ionization potentials (IPs). In these cases, the states of interest are the neutral GS and the core-cation GS. The XPS peak intensities were evaluated within the framework of the Dyson orbital formalism and the sudden approximation. This constitutes a very efficient approximation, that has been shown to be comparable with higher levels of theory.^52−54^ Vibronic effects were also included via the DHO, combining GS frequencies and normal modes with core-cation GS gradient (evaluated at the neutral GS minimum), and the simulated spectra normalized to match the most intense experimental band.

Scalar relativistic effects were taken into account, via a second order Douglass-Kroll-Hess Hamiltonian in combination with a generally contracted relativistic atomic natural orbital basis set (ANO-RCC).^55^ A density-fitting approximation, known as Cholesky decomposition,^56^ of the electron repulsion integrals has been used to speed up the calculation of two-electron integrals. The single state (SS) variant of the CASPT2 method was used for calculations of the ground states of the neutral and ionic species, whereas the multistate (MS) variant was used for core-excited states, unless specified otherwise (details in the SI).

A strategy to properly select the active space in a semiautomatic fashion that allows the progressive inclusion of a larger number of transitions is also reported. Additional computational details, such as the level of theory, the selected active spaces, the basis set contraction, the number of states considered, and a detailed description of the protocol employed, are reported in the SI.

## Results

**Figure 2:**
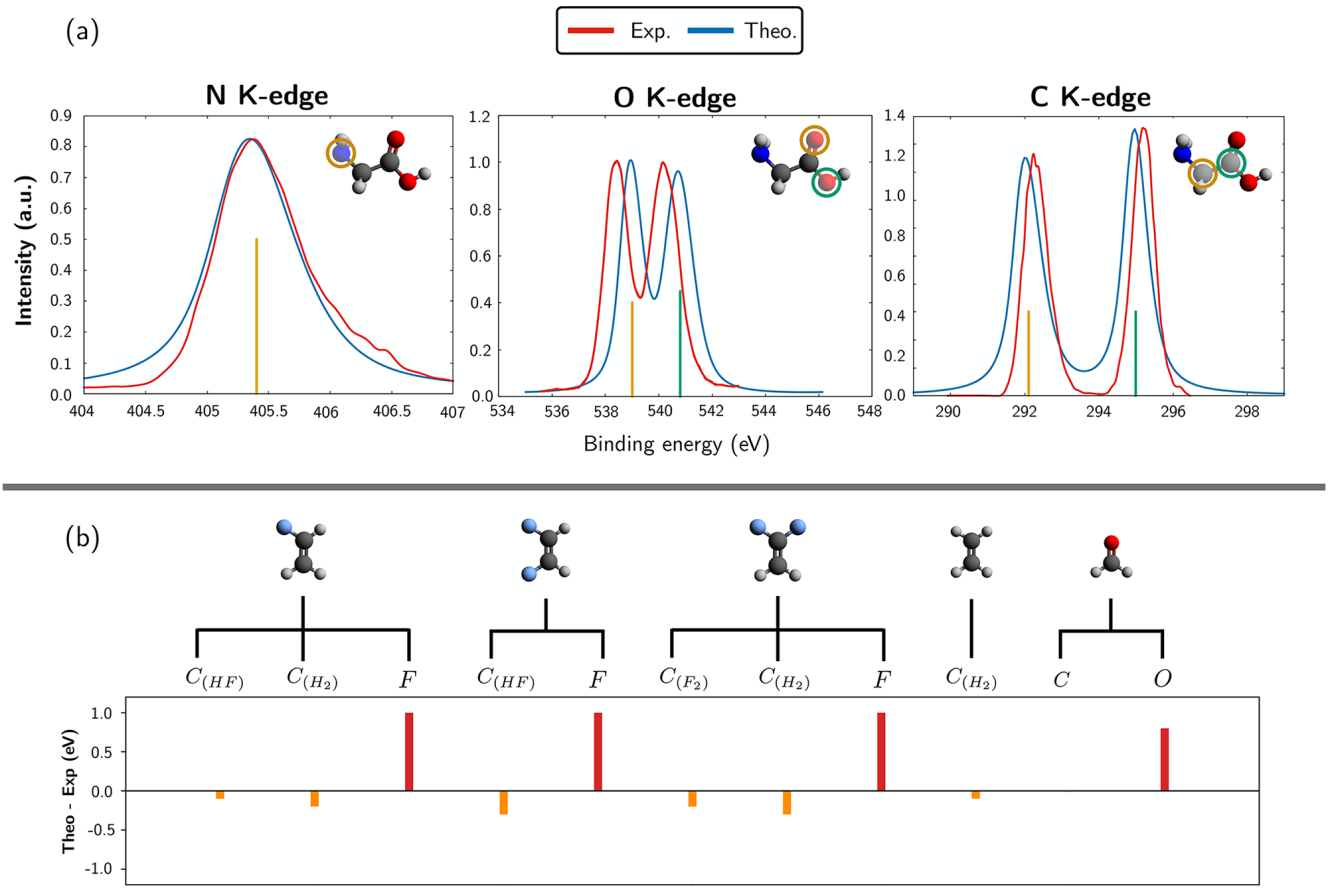
Simulation of XPS and IPs of second-row elements. (a) Theoretical (blue curves) and experimental^57^ (red curves) XPS spectra at the nitrogen, carbon, and oxygen K-edges for the glycine molecule. The contribution of individual cores to the various spectra is highlighted with sticks of different colors. No shift has been applied to theoretical spectra. (b) Difference between computed and experimental IPs for carbon and fluorine in a series of fluoro substituted ethanes/ethylenes as well as for carbon and oxygen in formaldehyde. The experimental IP values are taken from refs (58) and (59).

We have applied the RASSCF/RASPT2+DHO protocol to several molecular systems. Excited states are conveniently labeled with the pair of molecular orbitals which dominate the excitation process (the complete multiconfigurational states characterization is reported in the SI). To avoid ambiguity in the assignment of spectra exhibiting signals from more than one atom of the same type, core orbitals are labeled by the chemical formula of the nearest neighbor allowing unique identification. For example, in the glycine molecule (see Figure 2(a)), the two carbon centers were labeled as *C*_*O*_ and , respectively.

We note that due to the availability of experimental spectra, fluorinated ethylenes, glycine, and formaldehyde represent a common choice for benchmarking purposes. In fact, their XANES and XPS spectra simulations from single-reference methods have been reported in recent years.^16,39,42,43^ Nonetheless, the accuracy, robustness, and reliability of the multireference + DHO methodology to predict and reproduce energies, intensities, and line shapes of experimental spectra and the analysis of the results reported here are unprecedented.

### Simulations with the RASSCF/RASPT2+DHO Protocol: Sub-eV Accuracy from First Principles

#### XPS Spectra

Theoretical and experimental XPS spectra for N, C, and O K-edges of glycine, separated by ca. 100 eV from each other, are compared in Figure 2(a). The optimized glycine geometry of the most stable conformer out of the five identified by Miller and Clary^60^ was used and is sketched in the top right corner of the spectra. One observes a single peak at the N K-edge, centered at ca. 405.4 eV, due to the ionization of the 1s  core orbital; two peaks are observed for both the O and C K-edges, due to the different local chemical environments: in the former, the experimental *O*_*C*_ and *O*_*H*_ peaks are separated by ca. 1.8 eV, while in the latter, peaks related to  and *C*_*O*_ 1s ionization are 2.9 eV apart.

The agreement with the experiments^57^ is remarkable, not only in terms of the relative distances between the pairs of O and C peaks (identical to those measured) but also in terms of absolute energy (0.6 eV error for *O*_*C*_ and *O*_*H*_, 0.2 eV for  and *C*_*O*_, and negligible error in the case of ), line shape, and relative intensity of the peaks.

A comparison with CC3 and CCSD results of ref (43) (with aug-cc-pCVTZ basis set) reveals that at both levels the error in the energy gap between the pair of C and O peaks was of the order of ∼0.2 eV (slightly larger than the one reported here); for CCSD, a 1–1.5 eV absolute energy error is observed, which is instead strongly reduced in the case of CC3. Interestingly, the largest absolute energy error for CC3 is also observed for the O K-edge, a fact that will be analyzed again toward the end of the Discussion section.

Figure 2(b) shows the difference between theoretical (RASSCF/RASPT2) and experimental IPs for some fluorinated ethanes and for formaldehyde. Since XPS spectra are not available for these molecules, we only compare with the experimental IP values. We find the following:

- The errors on carbon IPs are always small, ranging from 0.01 to 0.4 eV, and the computed values are always underestimated.
- For fluorine and oxygen, the computed IPs are systematically overestimated by about 1.0 and 0.6 eV, respectively.

The systematic over/underestimation of the IPs for different atom types suggests the presence of a systematic error, that will be addressed in the Discussion section.

#### XANES Spectra

**Figure 3:**
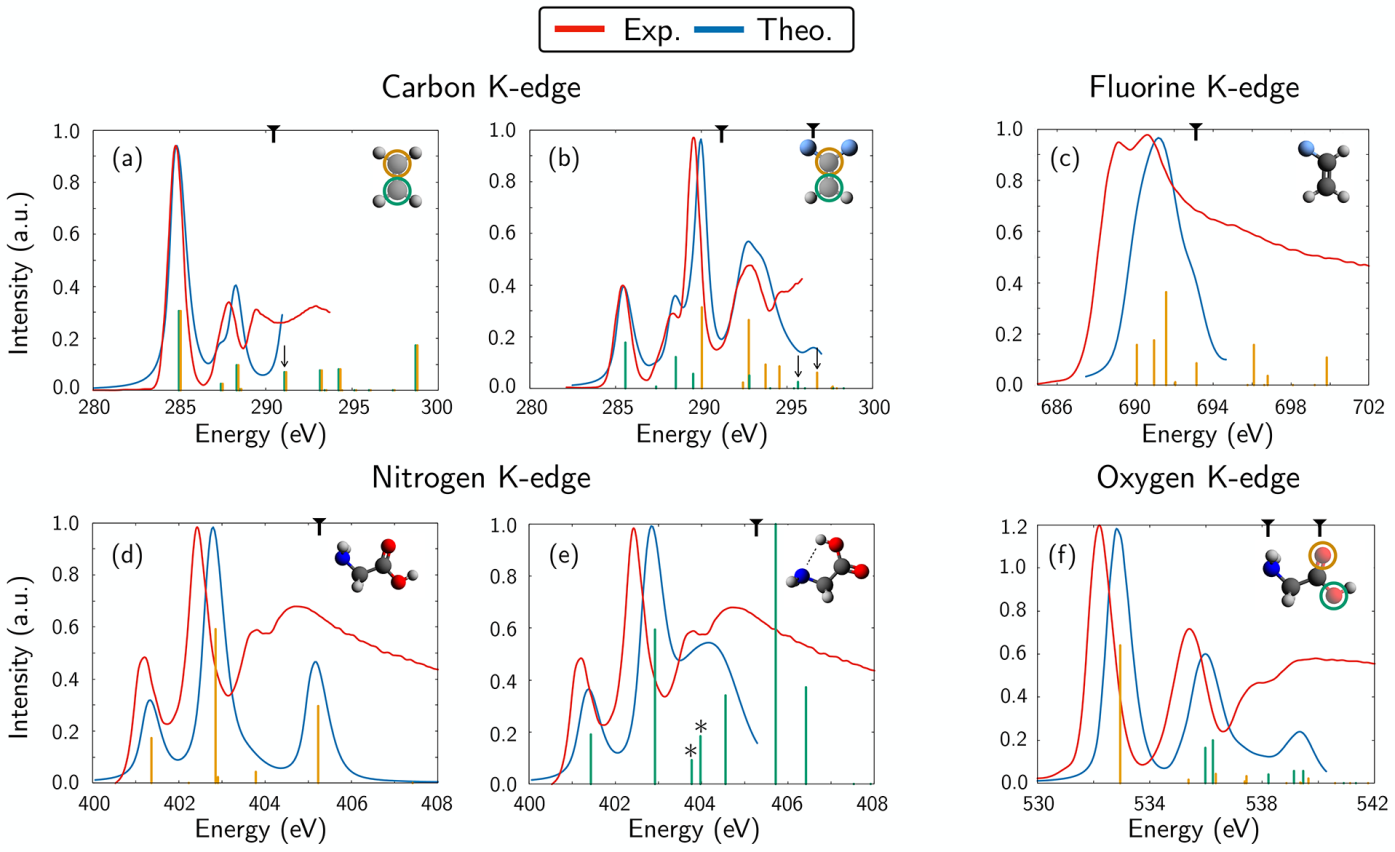
XANES spectra at the C, F, N, and O K-edges for various molecular systems: (a,b) C K-edge for ethane and 1,1-difluoroethylene, (c) F K-edge for vinyl fluoride, (d,e) N K-edge for glycine (two most populated conformers in gas phase, at 473 K), and (f) O K-edge for glycine (most stable conformer). The contributions of individual core transitions are highlighted with sticks of different colors (green and orange, respectively) when necessary, while the total spectra are depicted in blue. The red curve represents the experimental spectra from (a,b) ref (58), (d,f) ref (61), and (c) ref (58). The theoretical spectra are displayed with their line shape below the experimental IP threshold (indicated by the black mark on the top edge of the spectra), whereas above it they are reported only as sticks. Shake-up (or mixed core–valence) states have been highlighted with black arrows. In (e), transitions originating from the intramolecular hydrogen bonding of the cyclic conformer are highlighted with an asterisk. No shift has been applied to the theoretical spectra.

We present in Figure 3 XANES spectra at the C, N, F, and O K-edges (additional spectra are also reported in the SI in Section S7). Again, a remarkable agreement between the experimental and RASSCF/RASPT2+DHO theoretical spectra is found at the various edges, in terms of spectral position, relative intensity, and line shapes of the peaks. Computed transitions that lie above the ionization threshold (indicated by the black mark on the top edge of the spectra), which are thus characterized by shorter lifetimes and broader spectral features, have been reported as sticks. Shake-up features which appear in the simulated energy window have been highlighted in the spectra with black arrows (additional information about shake-up states configurations and comparison with their valence counterparts is reported in section S6 of the SI).

The carbon K-edge XANES spectra of ethane and 1,1-difluoroethylene are shown in Figure 3(a,b). Here, the sensitivity of the XPS and XANES techniques to the local/chemical environment is clearly demonstrated: the substitution of a hydrogen with a strongly electronegative fluorine alters the charge distribution over the nearby carbon, thereby increasing its 1s binding energy. This translates into a blue-shift of the fluorinated carbon 1s → π* transition which increases with the degree of fluorination. In particular, the main XANES peak experiences a blue-shift from ∼285 eV () through ∼287.5 eV (*C*_*HF*_) to ∼291 eV (). The unmodified carbon exhibits only a minor 0.5 eV blue-shift from its original position, a second-order effect due to the altered chemical environment of its first neighbor. A comprehensive analysis of a larger set of fluorinated ethanes is reported in the SI (see Section S7), where observed peak shifts are also rationalized in terms of symmetric and asymmetric fluorination. The same set of fluorinated ethylenes was studied in ref (16) at the coupled cluster, density functional, and static-exchange levels of theory. By comparing the highest level analyzed in ref (16) (i.e., CCSD with relativistic corrections and approximate treatment of triple excitations, employing a triple-ζ basis set with additional core-polarization functions for carbon) with the results presented here, and focusing on the core–valence 1s → π* excitations, one notices the following: absolute transition energies are required to be shifted by about 1–1.5 eV to match the experiment (while no shift is required here); the errors in the energy separation of the two 1s → π* peaks are within 0.1 eV (comparable with those found here); a large number of low-intensity Rydberg transitions were evaluated, contributing as a semicontinuum background in the spectra (while a lower number of states were considered here, which nonetheless capture most of the bright pre-edge signals); the oscillator strengths are similar to the RASSCF/RASPT2 ones; and the phenomenological line broadening of ∼0.124 eV does not properly capture the experimental line shapes (which is instead clearly reproduced here, by virtue of coupling the electronic transitions with the nuclear degrees of freedom via the DHO model). The agreement of the RASSCF/RASPT2+DHO approach with experiments demonstrates the accuracy of the simulation method, suggesting that it can be used to make predictions even when experiments are not available.

The XANES at the nitrogen K-edge of glycine in the gas phase is reported in Figure 3(d,e). Glycine is one of the smallest molecules that shows intramolecular hydrogen bonding.^60^ The interaction between the amino and the hydroxylic group gives rise to a controversial conformational equilibrium: five stable conformers have been reported. Here, we focus on the two most populated conformations in the gas phase (at 473 K), as shown at the top of Figure 3(d,e). Conformer A (Figure 3(d)) is linear and has been identified as the most stable one.^61^ Conformer B (Figure 3(e)) shows an intramolecular hydrogen bond, thus being termed “cyclic”. We computed XANES spectra of the A and B conformers and compared them with experiment. The red side of the XANES spectrum of both conformers is dominated by two intense peaks, separated by more than 1 eV, which our computations identify as transitions from the nitrogen 1s to two different mixed valence/Rydberg orbitals (1s → *V*/*Ry*). Moving toward the center of the spectral window, the main difference between the two conformers is located between 403.5 and 406 eV: in fact, the spectrum of A contains a single peak placed at 405.2 eV, belonging to a 1s → *V*/*Ry* transition, while not showing any intensity in the 403.5–404.5 eV region, where a peak is clearly visible in the experimental spectrum. In the spectrum of B, instead, the 405.2 eV feature disappears accompanied by the appearance of new peaks in the 403.5–404.5 eV window. These peaks are indeed identified as transitions to *V*/*Ry* orbitals, originating from the hydrogen bond (see * marks in Figure 3(e) and the orbitals depicted in Figure S17 of the SI). Interestingly enough, a similar role of the B conformer in determining the shape of the glycine XANES spectrum has not been reported in the literature, even if glycine has been studied in several theoretical works. In ref (43), for example, where simulations of XANES spectra at C, N, and O edges were performed at CC3 and CCSD levels of theory, it is claimed that the conformer effects would be small and difficult to detect experimentally. Besides the various approximations employed (no account of relativistic corrections and of vibrational contributions), the authors limited the conformer analysis to the O K-edge: they still found that the B conformer shows the largest spectral differences with respect to the A conformer, but they also argue that this effect would be small due to the low Boltzmann weight of the B conformer (10% at 300 K). In contrast, the analysis reported here considers the N K-edge, for which a new signal from conformer B is demonstrated to appear in an almost background free region; moreover, by considering the proper gas-phase glycine temperature (∼473 K), a larger weight of about 20% of the B conformer has to be taken into account (the MP2 Gibbs free energy difference of these two conformers at 473 K is about 6.535 kJ/mol). We also compared our results to those reported in ref (42), where a DFT based core-level spectrum of glycine with inclusion of MD based nuclear sampling was presented. The simulated spectrum at the A conformer minimum (i.e., prior to the nuclear sampling) reveals the presence of significant intensity also in the 403.5–404.5 eV region, where we demonstrated that the B conformer should mainly contribute. In the SI (see Section S8), we report a calculation performed on the glycine conformer A restricting the configuration state function space to a single electron excitation: interestingly, the very weak transition around 403.8 of Figure 3(d) becomes extremely intense. In this last calculation, the state is dominated by two single excitations with large coefficients, while in the complete calculation (where up to four excitations were allowed), many multiple-excitation contributions appear, with a concomitant reduction of the single-excitation character of the transition. The protocol is accurate enough to precisely pinpoint the signals specific to the hydrogen bond in the N K-edge, and the multiconfigurational nature of the RASSCF/RASPT2 method is shown to be paramount in order to properly describe the intensity of the observed signals and thus elucidate the origin of the measured XANES spectral features.

Finally, the oxygen and fluorine K-edge XANES spectra are shown for glycine and vinyl fluoride in Figure 3(f) and (c), respectively. The oxygen K-edge of the glycine molecule (in the most stable conformation) is characterized by two main peaks: one placed at ∼533 eV and the other placed at ∼536 eV, labeled, respectively, as the  transition and as the combination of the  with a  transition. Experimental line shapes and relative intensities are well reproduced, while transition energies are blue-shifted by ca. 0.5 eV with respect to experiment, as it was the case for the XPS simulation results previously reported. For the fluorine K-edge of vinyl fluoride, we observe a single broad band centered at 691 eV with slightly visible shoulders to the red and to the blue, originating from multiple transitions, while the experiment seems to indicate two equally intense close-lying transitions at a lower energy of about 1 eV. Also here, the 1 eV deviation is consistent with the one reported for the computed IP values in the previous sections, reinforcing the notion of a systematic error in the calculations, that will be discussed in the next section. A comparison with the O K-edge CC3 and CCSD simulations reported in ref (43) reveals a similar orbital labeling of the reported transitions, which should be shifted about +0.10 eV and −1.76 eV, respectively, at the two levels. The intensity of the CC3 second oxygen band is largely overestimated, due to nearly degeneracy of the two states that contribute to the band intensity; the degeneracy is instead lifted (and the spectrum slightly improved) at the CCSD level, that nonetheless presents an error in the relative distance between the two bands.

## Discussion

The spectra presented in the Results section show a remarkable agreement between simulation and experiment, demonstrating the substantial performances of the RASSCF/RASPT2+DHO approach, also in comparison with other levels of theory. To gain further insight, we focus here on the systematic under/overestimation of the transition energies (i.e., the slight red-shift observed for C K-edge IPs and the blue-shift of O and F K-edges XANES) as well as some subtle discord in the line shapes (in the case of vynil fluoride F K-edge). Given the accuracy of the RASSCF/RASPT2 quantum chemistry data, major theory-experiment discrepancies should be ascribed to assumptions/approximations inherent in the DHO model and the signal simulation. In this respect, the DHO model neglects the different energy profiles of the potential energy surface (PES) of the various electronic states (the Duschinsky effect) and makes the strong approximation that all states of interest are described by identical harmonic wells, with the same frequencies along a unique set of normal mode coordinates (which are thus computed only for a single electronic state, typically the GS); these identical PESs only differ by their equilibrium positions. Furthermore, the modes are assumed to be uncoupled, and possible anharmonicities are not accounted for. Finally, the dependence of the transition dipole moment on the nuclear coordinates (the Herzberg–Teller effect) is also neglected (the Condon approximation). We demonstrate that these approximations have a stronger impact on core transitions as compared to valence transitions and can be used to explain the observed discrepancies between theory and experiment.

### a The Duschinsky Effect

It is generally understood that, upon removal of an electron from a core orbital, the shielding of the nuclear charges reduces dramatically (or, which is the same, the effective nuclear potential increases), resulting in a stronger attraction of the outer electrons and in a compression of the orbitals toward the nucleus. This will produce a sudden reorganization of the charge density, affect the electron–nuclear coupling, induce a molecular structure modification (bond shortening/elongation), and change the bond force constants and thus the normal-mode frequencies. These effects lead to a breaking of the identical PES approximation (to a different extent for different atoms) and eventually impact the spectroscopic signals, in terms of peak position, intensity, and line shape.

### b The Herzberg–Teller Effect

Core excitations are highly localized in space. For a transition labeled as 1s → *a*, where *a* denotes a generic arrival molecular orbital, the intensity depends on the extent of the spatial overlap between 1s and *a*. The more localized orbital *a* is on the core-excited atom, the more intense the transition will be. Given the locality of the transition, it is clear that bond elongation/shortening during vibrational motion can strongly affect the intensity of the signals and therefore that non-Condon effects are expected to play a non-negligible role.

Below we give some additional insights into these effects, enlightening, at the same time, the crucial ingredients that should be considered to properly account for the electron–nuclear coupling in the case of core excitation/ionization. In particular, a qualitative orbital-based criterion is provided to explain the structural relaxation that follows the removal of a core electron, in a unitary framework that comprises both core-ionization and core-excitation processes.

Let us consider the valence orbitals of two bound atoms: from the point of view of molecular orbital theory, the bond strength can be captured by the extent of the overlap between the atomic orbitals of the two centers. This quantity mainly depends on three (atomic orbitals) parameters: (i) the phase; (ii) the superposition of the angular part; and (iii) the superposition of the radial part. In the case of two atomic orbitals interacting in phase, upon excitation of a core electron from one of the two centers, the angular part remains unaltered, but the radial part of the core-excited atomic orbital is affected due to the increase in the effective nuclear potential: this leads to the compression of the atomic orbitals toward the nucleus of the core-excited center. Qualitatively, the core ionization corresponds to a sudden substitution of the considered atom with the one at its right in the periodic table (*Z* + 1 approximation); the atomic orbitals of the core ionized atom will therefore resamble those of the *Z* + 1 neutral atom. The effect of the orbital compression on the bond strength depends on the involved atom types. Moreover, the degree of compression varies for core-ionization (as in XPS) and core-excitation (as in XANES) processes. We consider the C–F bond of the fluoroethenes systems as a testbed, highlighting the impact of the described effects on the spectra (see also Section S9 of the SI, where a detailed analysis of bond length changes upon C and F ionization/excitation is provided for the various fluoroethenes).

### Core Ionization

**Figure 4:**
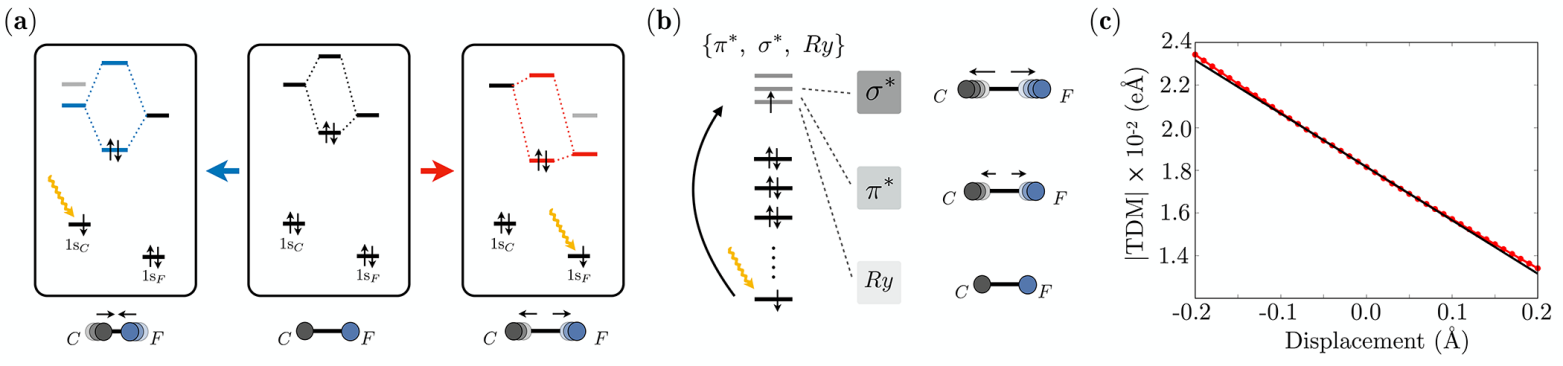
(a) Orbital relaxation and bond strength variation upon core ionization of either carbon or fluorine in the C–F bond. When 1s_*C*_ is excited (left panel), the equilibrium distance becomes shorter leading to higher vibrational frequency; vice versa, when 1s_*F*_ is excited (right panel), the equilibrium bond distance becomes longer leading to lower vibrational frequency; (b) bond length (and strength) variation as a function of the antibonding orbital shape, in which the core-excited electron is relocated (in the case of, e.g., XANES). The effect ranges from negligible to strong in going from *Ry*, through π* to σ* type orbitals. (c) Profile of the transition dipole moment module along the C–F stretching mode for the lowest energy 1s_*F*_ → π* transition in vinyl fluoride (red dotted curve) and its tangent at 0 displacement (black curve). Note the slight deviation from linearity at the largest displacements.

The level scheme of Figure 4(a) summarizes the effects that the compression of the orbitals causes on a bond between two atoms with different *Z* numbers (heteronuclear case): on the one hand, if the atom with the lower *Z* is core-ionized, the compression of the energy levels can bring its orbitals’ energy closer to those of the other atom and can therefore strengthen the bond (Figure 4(a), left panel); on the other hand, if the core ionization involves the orbitals of the higher Z atom, the lowering of its orbitals’ energy can decrease the extent of the overlap with the other atom, therefore decreasing the bond strength (Figure 4(a), right panel). [We note that similar considerations can only be applied for atoms of the same row. Moreover, third- and higher-row elements also require special attention, because a much stronger relativistic coupling has to be taken into account.] When atoms with the same *Z* are considered (homonuclear case), the core excitation always leads to bond weakening. We note that core vacancy induced weakening of the bonds can be extremely pronounced and promote the dissociation of the molecule (a scenario which is rarely observed in valence excitations).

Considering *C*_*F*_ of the fluorinated ethylenes, the core ionization lowers the energy of its 2*p* orbitals increasing the overlap with the 2*p* orbitals of fluorine, thus giving rise to a stronger σ bond in the core-ionized state, accompanied by a *C*–*F* bond shortening (Figure 4(a)), which consequently leads to an increasing of the *C*–*F* stretching frequency. On the other hand, the core ionization from *F* drastically lowers the energy of its 2*p* orbitals decreasing the extent of the overlap with the carbon 2*p* orbitals. This effect gives rise to a weaker σ interaction which ultimately leads to the breaking of the *C*–*F* bond and the dissociation of the fluorine atom from the molecule (see Section S9 of the SI). These electronic structure changes lead to the breaking of the identical PESs approximation: in the case of *C*_*F*_, the core-ionized state will be characterized by the above-mentioned increase of the *C*–*F* vibrational frequency, giving rise to a positive zero point energy (ΔZPE) contribution. In the case of F, the core-ionized state dissociative potential cannot even be described by the harmonic approximation. By taking these effects into account, it is possible to rationalize the observed shift from the experiment in the IP simulations of the fluorinated ethanes. Figure 2(b) displays that the simulated carbon IPs are always red-shifted from the experiment, while the fluorine IPs show a constant blue-shift of about 1 eV from the experiment. The consideration of the ΔZPE would blue-shift the carbon XPS toward a better agreement with the experiments; for the fluorine IPs, the drastic lowering of the energy along the *C*–*F* dissociation coordinate will affect the spectra introducing a significant red-shift. This shift, ultimately related to the *C*–*F* dissociation energy, can be reasonably assumed to be constant for all the fluorinated molecules considered, therefore explaining the systematic absolute energy deviation between our simulations and the experimental IPs.

### Core Excitation

Looking at core-excited states requires considering the additional effect of the radial and angular distribution of the antibonding orbitals in which the core-excited electron is relocated, i.e., of their *shape*. Since different types of antibonding orbitals exist, three different situations need to be considered: (i) the filling of a π* orbital decreases the bond order, producing a moderate relaxation of the considered bond; (ii) the filling of a σ* orbital decreases the bond order, producing a strong relaxation; and (iii) the filling of a *Ry* or mixed valence/*Ry* orbital produces an almost neutral effect on the bond order, which is reflected in the retention of the considered bond length (see Figure 4(b)). Let us complete the example of the *C*–*F* bond considering both the removal of one electron from a core orbital and its relocation into higher-lying antibonding orbitals (that takes place, for example, in XANES). If the σ* (or the π*) orbital is filled with an electron from the fluorine core orbital, the two effects cooperate, further promoting the dissociation: in vinyl fluoride, the different nature of the arrival orbitals (σ* and π*) of the first few F pre-edge states will produce a different profile of the respective dissociative PESs and might be the origin of the inability to correctly reproduce the experimental line shape (see Figure 3(c)). If, at variance, the electron comes from the core orbital of the carbon, the two effects cancel each other out to a large extent. This subtle balance is at the origin of the satisfactory performance of the DHO when applied to the C K-edge of the reported molecules. The latter example shows that the core-(half)emptying and the antibonding orbital population may contrast each other, and the net effect depends on which of the two contributions prevails.

The combination of effects discussed so far, i.e., the bond-order modulation upon core hole formation (either weakening or strengthening) and population of antibonding orbitals (weakening), can give rise to a variety of possible scenarios, depending on the nature of the considered excited state and on the complete bond network that surrounds the core-excited center. The described bond relaxation effects can be quantified by the DHO itself: even if we demonstrated its intrinsic limitations in the ability to properly describe the electron–nuclear coupling in core excitations/ionizations, it can still signal a significant change in the excited state geometry in terms of mode-specific reorganization energies. These vary from very small to extremely large according to the nature of the core excitation/ionization. A table that summarizes the most significant cases is reported in the Supporting Information (Section S9).

In passing, we note that this simple criterion is capable of explaining trends observed in previous XPS (experimental and theoretical) studies, such as the bond shortening and bond elongation in CO^62,63^ upon C and O core ionization, respectively, and the bond elongation in, e.g., O_2_^64^ and N_2_.^65^ These results were often rationalized in terms of electronic distribution reorganization upon core ionization, employing the ESCA potential model for chemical shifts.^63,66^ The orbital-based criterion given here summarizes the core-hole formation effect in a unique framework for core excitation and core ionization, and it explains the observed bond changes of the analyzed molecular systems.

Finally, Figure 4(c) shows the profile of the transition dipole moment module of the lowest energy 1s_*F*_ → π* transition in vinyl fluoride, along the C–F stretching mode. If the Condon approximation holds, the TDM would remain constant, while it clearly shows a marked mode dependence. The figure also displays the tangent at 0 displacement (black curve), which makes it easier to appreciate the slight deviation from linearity at larger displacements.

### Simulations beyond the DHO Approximation: Toward a Quantitative Agreement

Although the qualitative picture drawn above is able to describe the direction of the shift observed in the IPs, the full quantitative treatment (of nondissociative core-excited/ionized states) requires a further (modeling) step to be undertaken. This further step is here described for the simulation of the formaldehyde C K-edge, where the Duschinsky rotation^39,67−69^ of the modes was taken into account. The spectra computations were performed employing the FC-classes code.^70^ Additional details are provided in the SI (see Sections S5 and S10).

**Figure 5:**
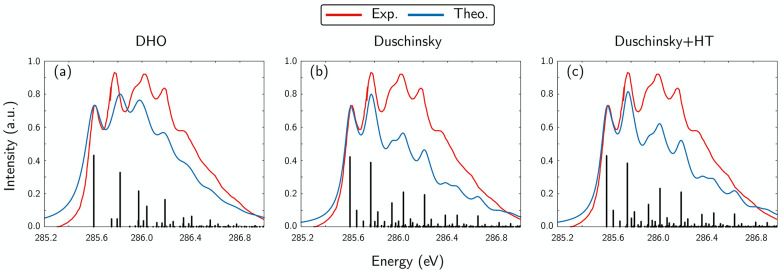
C K-edge XANES spectrum of the 1s → π* transition in the formaldehyde molecule at different levels of theory: a) DHO, b) including Duschinsky rotations, and c) including both Duschinsky and Herzberg–Teller effects. The theoretical spectra were shifted by 0.1 eV to allow a better comparison with the experimental vibronic progression (red curve) from ref (59).

The 1s_*C*_ → π* experimental XANES spectrum^59^ shows a well resolved vibronic structure, as reported in Figure 5, where a comparison of three levels of theory is also presented. One notices that the DHO model captures the essential vibronic structure (dominated by the C=O stretching and by the C–H symmetric stretching, with 1753 and 2970 cm^–1^ frequencies, respectively), while lacking a quantitative agreement. Upon introducing the Duschinsky effect, the improvement in the description of the experimental vibronic peaks is significant (see Figure 5(b)). In particular, one notices the splitting of the DHO third band in a pair of subpeaks, clearly observed also in the experiment. Herzberg–Teller effects were eventually accounted for (assuming a linear dependence of the transition dipole moment on the nuclear coordinates), leading to a slight additional improvement of the relative intensities of the vibrational bands. A further improvement may be obtained going beyond the first order expansion of the transition dipole moment in the space of the nuclear coordinates.

A similar analysis was performed at the (EOM-)CCSD level of theory in ref (39), where Duschinsky mixing was also taken into account. The modes that mainly contribute to the spectrum are the same reported here, while the simulated spectrum shown here in Figure 5 resembles more closely the experimental one (see in particular the red shoulder of the third peak that is captured at the present level once Duschinsky mixing is accounted for). The differences between the simulated and observed progressions in ref (39) were ascribed to a nonperfect description of the (EOM-)CCSD ES PES, indicating that inclusion of triple and quadruple corrections might be important here. Indeed, the authors report a C=O equilibrium bond length of 1.202 Å in the GS and of 1.266 Å  in the core-ES, while we found these to be 1.213 and 1.293 Å  (at MP2 and RASPT2 levels), respectively, in better agreement with the experimental values of 1.207 and 1.316 Å  estimated by Remmers and co-workers.^59^

We also performed computations for the formaldehyde oxygen K-edge XANES, focusing on the 1s_*O*_ → π* transition. Here, the DHO fails to describe the proper position of the band, which is again blue-shifted with respect to the experiment, as the molecular structure lies on an excited state transition state. Notably, this observation is consistent with the fact that the largest absolute energy error for formaldehyde was observed at the O K-edge also at the CC3 level in ref (43). Therefore, we note that the systematic blue-shift of the O K-edge simulated bands (also previously reported for the glycine IPs and XANES bands) can be ascribed to the excited state pyramidalization (i.e., breaking of the planarity) of the CO bond in all of the studied cases. Indeed, treatment of the vibronic coupling with the DHO model is incapable of describing such a mode, as it was unable to describe the dissociative PESs at the F K-edge.

### Simulations toward Larger Molecular Systems: From Main Features to Spectrum Completeness with a *Divide and Conquer* Approach

RASSCF is not a black-box approach: it requires the active space to be properly tailored to the problem at hand. In the following, we argue that this nontrivial task can be actually turned into an advantage in the case of core-excited calculations.

The active space for core excitation/ionization must include the core orbital(s) of interest: these are placed in the subspace onto which the HEXS projection scheme is applied; virtual orbitals decide which core states are going to be described in the RASSCF/RASPT2 calculation: these are to be chosen by the user in a deliberate fashion, having in mind that a larger set of virtual orbitals assures a completeness of the spectrum; occupied valence orbitals are also added to provide a better description of electron correlation, as well as shake-up states. Often it suffices to include the highest occupied valence orbitals. In case the core excitation takes place in a molecule residing in an electronically excited state (as it happens in UV-pump X-ray probe schemes, such as TR-XANES), the relevant occupied orbitals should be chosen to properly describe valence- and core-excited states, as well as for account for electron correlation.

In what follows, we show a) how to design a minimal active space that allows capturing of the main pre-edge signals while minimizing the computational cost and b) an algorithm to systematically account for signals from different virtual orbitals while maintaining the cost of the computation. In both cases, we focus on the proper selection of the virtual orbital set to be included in the active space, employing 1,1-difluoroethene at the carbon K-edge as a suitable testbed. We did not impose any symmetry to the orbitals, to keep the discussion general.

**Figure 6:**
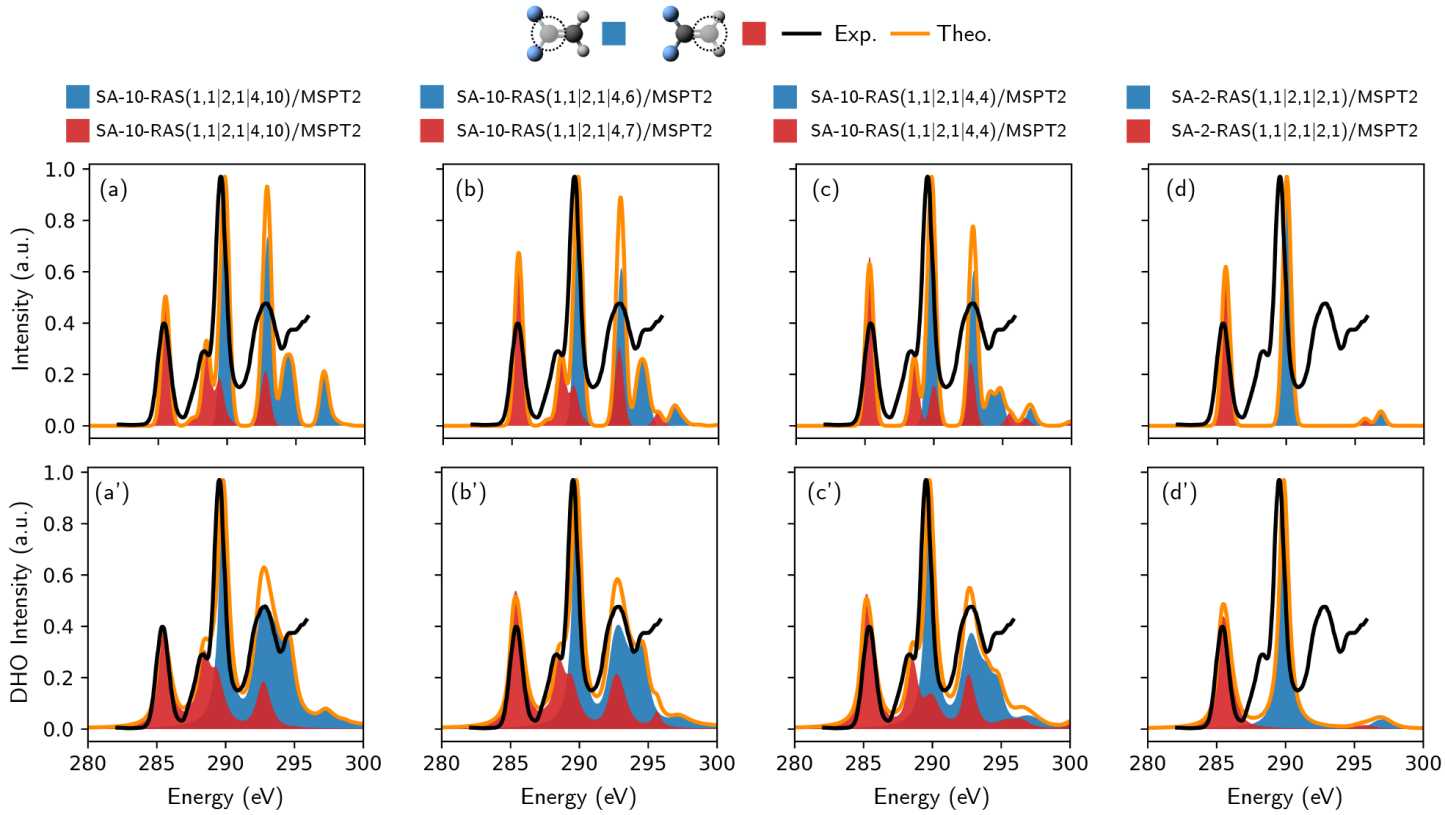
C K-edge XANES spectrum of 1,1-difluoroethane simulated with active spaces of decreasing sizes. In the first row ((a)–(d)), the line shape is modeled by the application of a phenomenological Gaussian broadening, while in the second row ((a′)–(d′)), the vibronic coupling was taken into account within the DHO framework. Spectra (a) and (a′) represent the largest active space, while the active space (RAS3) size is progressively reduced from left to right. The reference experimental spectrum (taken from ref (58)) is displayed in black. The theoretical results for different carbon atoms are highlighted with different colors, while their sum, i.e., the total theoretical spectrum, is shown in orange. No shift has been applied to the theoretical spectra.

By looking at the character of the 1,1-difluoroethene core-excited states (section S4 of the SI), one realizes that the HOMO orbital suffices to describe all the shake-up states; moreover, the missing correlation, due to the exclusion of other occupied orbitals, can be recovered by the PT2 procedure, which is stable due to the presence of the HOMO in the active space. Note that for a generic system, this fact is not known a priori: in that case, one should test the minimal number of occupied orbitals that capture the important shake-up states (that are typically the same that describes the first few valence excited states) and that make the PT2 procedure stable. Figure 6 (to be read from left–the largest active space–to right–the smallest active space) shows that by systematically reducing the number of virtual orbitals in the AS to the limit of containing only the π* orbital, one can still recover the main pre-edge signals with an extremely high accuracy. The role of the excluded orbitals is 2-fold: be potential “arrival orbitals” for the 1s excited electron (corresponding states will be bright if these orbitals possess a significant overlap with the 1s) and/or for possible multiple excitation processes (that can contribute to determining the signal intensity as seen in the results reported above). Even with the minimal active space containing the core, the HOMO (π) and the LUMO (π*) orbitals, the strongest pre-edge signals (π*-resonances), are nicely described in terms of energy positioning, relative intensity, and line shape (which also suggests that gradient calculations are stable against active space reduction).

Next, we present an algorithm capable of iteratively increasing the number of core excited states described while keeping the computational cost low. The algorithm leverages on the fact that the “gross” description of a bright transition is mainly given by single-electron configurations, with smaller contributions from two- and higher-electron contributions. This description is generally accurate for core excitations. The algorithm works as follows:

1. Choose a minimal active space, consisting of the core orbital, a small number of occupied valence orbitals (typically only the HOMO), and a small number of relevant virtual orbitals (typically the LUMO). We call this active space minAS;
2. Choose a small number *n* of additional virtual orbitals (*n* should not be exceedingly large, so to keep the cost of the computation low) to be added to RAS3 at each step. The AS is now minAS+*n*V_1_ (with the 1 subscript identifying the current iteration step);
3. Perform a RASSCF/RASPT2 computation of the core excited states (followed by a GS computation) within the minAS+*n*V_1_ active space, as well as a vertical gradient computation at the same level of theory (if desired);
4. Rotate the nV_1_ orbitals outside of the active space, lock them outside of the AS, and allow for a new set (*n*V_2_) of virtual orbitals to be included in the AS;
5. Iterate steps 3. and 4.; at every iteration step a new total spectrum is obtained as the sum of the spectra produced in the previous steps;
6. The iterative procedure is continued until convergence of the signals: as more and more virtual orbitals (with higher energy) are considered, the new signals will start to appear outside of the considered energy window, indicating that convergence is reached in the window of interest.

A final note concerns the fact that some transitions (e.g., 1s → LUMO and the associated shake-up state) will be recomputed at every iteration and need therefore to be removed prior to the summation of the spectra over the iteration steps.

**Figure 7:**
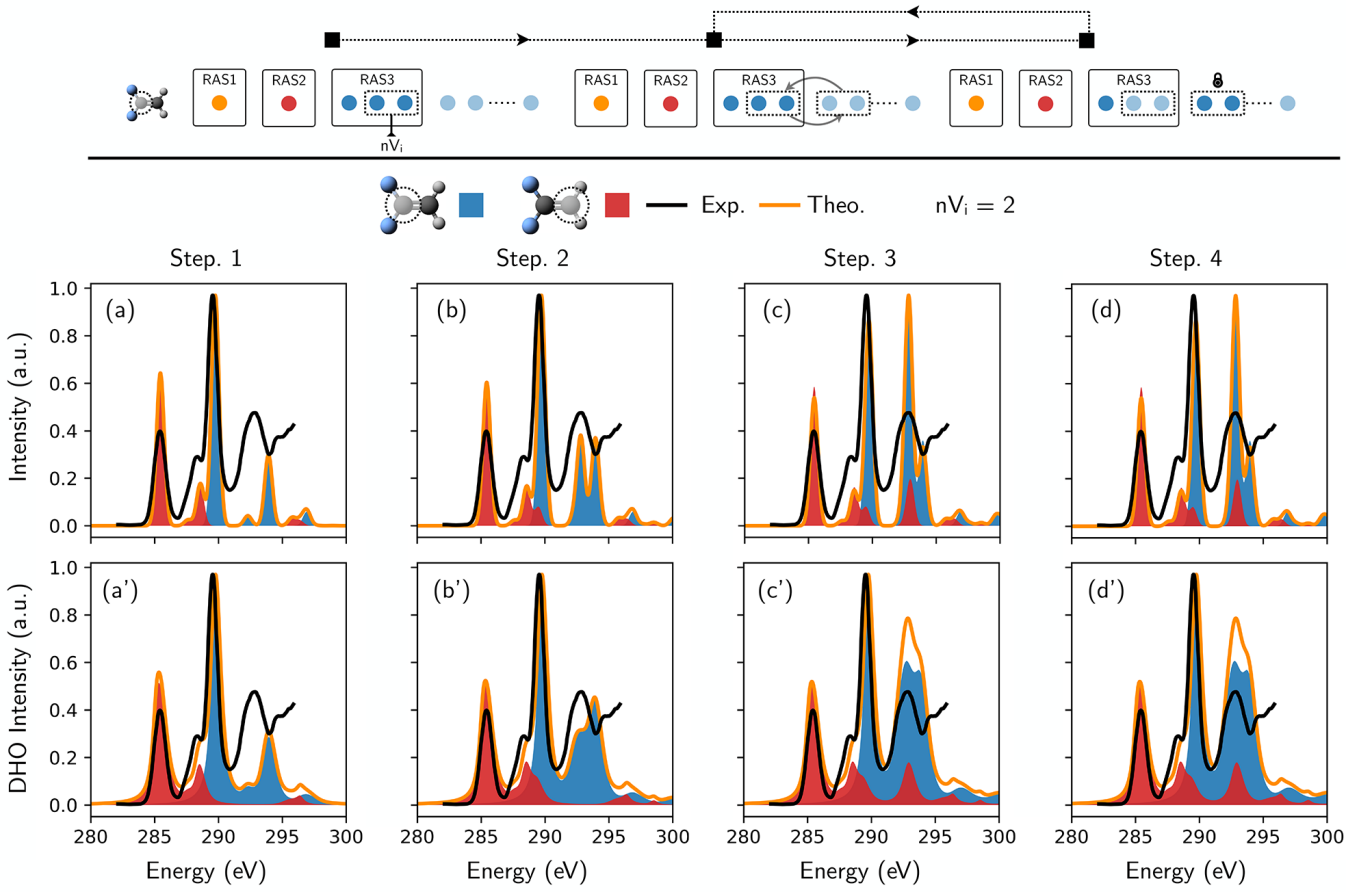
C K-edge XANES spectrum of 1,1-difluoroethane simulated with an automatic selection of a series of active spaces designed to account for a large number of signals at a reduced computational cost. *Top*: schematic description of the key steps of the algorithm. *First row* (a–d): spectra obtained at an increasing number of the algorithm iterations. The line shape was modeled applying a phenomenological Gaussian broadening. *Second row* (a′–d′): Same spectra modeled taking into account also the vibronic coupling. The reference experimental spectrum (taken from ref (58)) is displayed in black. The theoretical results for different carbon atoms are highlighted with different colors, while their sum, i.e., the total theoretical spectrum, is shown in orange. No shift has been applied to the theoretical spectra.

In Figure 7 (*top*), we give a pictorial representation of the *divide and conquer* approach described above, that allows exploration of a large region of the virtual orbitals’ space, with a sequence of low-cost serial computations (linear scaling vs exponential scaling). The larger the number *n* of virtual orbitals considered at each step, the better the description of static correlation will be. One should not decrease *n* too much, as some states can be described by the linear combination of many configuration state functions. Note also that the present approach ignores possible interactions between states computed in different iteration steps. Fortunately, if one can keep the number of occupied orbs reasonably small (e.g., core+HOMO), one could afford to increase the size of the virtual orbitals chunks to a dozen.

Application of the algorithm to 1,1-difluoroethane, employing an orbital chunk size of *n* = 2 is again reported in Figure 7 (*bottom*). Our results show a clear convergence of the spectral signals after 3 iterations, and by the comparison between the outputs of the various iteration (Figure 7(a)–(d)), it is possible to appreciate how the agreement between theory and experiment is increased at each step. Figure 7(a′)–(d′) also demonstrates the robustness of the gradient calculations (and therefore of the DHO model of vibrionic coupling) in each step. One notices that the main discrepancy between the converged spectra and the higher level spectra of Figure 6(a)/(a′) resides in the description of the relative intensity of the transitions, a side effect that can be reduced by enlarging *n*. It is thus clear that *n* represents the degree of freedom to obtain the best compromise between accuracy and computational cost.

## Conclusion

We have presented a general multireference based method for the calculation of core-excited/core-ionized states, that combine the RASSCF/RASPT2 quantum chemistry method with the displaced harmonic oscillator model to account for the vibronic coupling. Applications were made for the calculation of XANES and XPS spectra of the K-edges of second-row elements carbon, nitrogen, oxygen, and fluorine. The method represents a valuable addition to the state-of-the-art approaches (mostly single-reference in nature) and is shown to be highly accurate, reliable, efficient, and robust in reproducing experimental spectra of organic molecules exhibiting the following:

a) multiple K-edges, demonstrating its remarkable accuracy independent of the atom type;
b) a varying degree of functionalization, highlighting its sensitivity to the local/chemical environment by reproducing chemical shifts;
c) several coexisting conformers, evidencing its capacity to resolve subtle effects such as intramolecular hydrogen bonding and the importance of considering the multiconfigurational nature of some transitions;
d) a vibronic structure in the spectra, demonstrating its ability to accurately describe the electron–nuclear coupling.

Insights into the results have been given, the main deviations with respect to experiment have been explained, and improvements toward a quantitative agreement by incorporating Duschinsky and Herzberg–Teller effects have been discussed. The method is readily applicable to simulate K- and L-edge spectroscopy of third-row elements and beyond, as well as transferable to systems of medium/large size, for which an automated scheme for systematically increasing the number of considered transitions has been presented. The protocol can be customized to account also for the manifold of valence states and transition dipoles between and within valence and core-excited states, facilitating the simulation of a large variety of linear and nonlinear spectroscopic techniques that employ multiple optical and X-ray pulses.

Finally, the accuracy of the method puts it in the position to serve as a benchmark for existing and future theoretical approaches.

## Supporting Information Available

Spectra simulations, computational protocol: additional information, level of theory of computations, core-excited/ionized states lifetime, active spaces and multiconfigurational character of core-excited states, normal modes, shake-up transitions, XANES of fluorinated ethanes, glycine: single-configuration vs multiconfigurations, C–F example, Duschinsky and Herzberg–Teller (PDF)
