# Supplementary material for: Soft X-ray Spectroscopy Simulations with Multiconfigurational Wave Function Theory: Spectrum Completeness, Sub-eV Accuracy, and Quantitative Reproduction of Line Shapes
Source: J Chem Theory Comput. 2022 Jan 24;18(2):1003–16. doi: 10.1021/acs.jctc.1c00566 (PMC8830047; doi:10.1021/acs.jctc.1c00566)
Supplement: Supplementary file 1 — ct1c00566_si_001.pdf [file ct1c00566_si_001.pdf]

Supporting Information

for

Soft X-ray spectroscopy simulations with multiconfigurational wavefunction theory: spectrum completeness, sub-eV accuracy and quantitative reproduction of line shapes

Francesco Montorsi,[†] Francesco Segatta,[*,†] Artur Nenov,[†] Shaul Mukamel,[‡] and Marco Garavelli[*,†]

†*Department of Industrial Chemistry "Toso Montanari", University of Bologna, Viale del Risorgimento, 4, 40136 Bologna, Italy*

‡*Department of Chemistry and Department of Physics & Astronomy, University of California, Irvine, California 92697-2025, USA*

E-mail: francesco.segatta2@unibo.it; marco.garavelli@unibo.it

S1 Spectra Simulation

Both XANES and XPS spectra were obtained in the sum-over-states approach, as the Fourier transform of the the first-order response function given by[1,2]

$$R^{(1)}(t) = \left(\frac{i}{\hbar}\right) \theta(t) \sum_{e} |\boldsymbol{\mu}_{eg}|^2 \exp\left[-i\left(\omega_{ge} - i\tau_{ge}^{-1}\right)t - 2g_{ee}(t)\right] + c.c. \tag{1}$$

where $\sum_e$ run over the core-excited (core-ionized) states and ω_{ge} is the energy gap between these and the ground-state for XANES (XPS) spectra. τ_{eg} indicates the finite lifetime of the core-excited/core-ionized state (that give rise to the homogeneous broadening of the spectral lines), while $g_{ee}(t)$ is the so called line-shape function, which encodes the coupling of the electronic states with the intra-molecular vibrations (and the environment) in terms of displacements of ES equilibrium position along the normal modes coordinates. These displacements can be computed with the vertical gradient approach, implemented in iSPECTRON,[2] i.e. by projecting energy gradients of each excited (cationic) state e onto the GS normal modes.

S2 Computational protocol: additional information

In the following we present the details of the computational protocol used for the computation of XANES and XPS spectra. A schematic representation of this protocol is provided in Figure 1 of the manuscript. The key steps are to get the fundamental quantum chemistry quantity are:

1. MP2 geometry optimization on the molecular ground state;

2. Hartree-Fock (HF) single point calculation to select initial orbitals for the active space. Localized 1s orbitals on the atoms of interest are always considered, as they facilitate the interpretation of core-excited signals from different atoms of the same type. If delocalized canonical core orbitals are obtained, suitable linear combinations of them are performed, and the wanted localized core-orbitals obtained. Note that the use of localized core-orbitals may lower the orbitals symmetry (that can be applied to speed up the calculations). For the molecular systems here considered, a C_s point group symmetry was employed in all the calculations.

3. Active space construction, consisting of three different sub-spaces:

   (a) RAS1: one core orbital is placed herein and kept frozen to avoid orbital rotation; the upper limit of holes is set to 1;

   (b) RAS2: occupied (at SCF level) valence orbitals of prior importance to describe static correlation are placed herein;

   (c) RAS3: virtual orbitals that could give rise to bright transitions are placed herein and the upper limit excitation is set to 4.

   For the systems considered in this work, we included in RAS2 π and n type orbitals (when present) since they can give rise to bright shake-up transitions. In the RAS3 sub-space, only a few orbitals were manually inserted, namely the π^* type orbitals, which

are expected to be involved in the brightest states. The remaining large set of mixed valence/Rydberg orbitals found there, is optimized automatically by the RASSCF routine to properly describe the remaining states. In some cases, to avoid the rotation of some valence orbital outside of RAS2, these were kept frozen.

4. RASSCF calculations are performed separately for:

   (a) the GS wave-function and energy (state specific RASSCF computation);

   (b) the core-excited states manifold (state average RASSCF calculation); in order to force the formation of the core-hole configurations, the HEXS projection technique (implemented in OpenMolcas) is applied to the RAS1 state.

   For the molecular systems considered here, a C_s point group symmetry was always employed.

5. RASPT2 calculation on the top of both ground and excited state RASSCF wave-funcions. In this step, a technical (but important) issue has to be considered: the number of frozen orbital has to be set to zero. It is in fact typical, to reduce the computational cost, that the RASPT2 routine avoid to consider the orbitals placed at low indices in the list of molecular orbitals (were core orbitals typically lie). Nevertheless, the rotation of a core orbital in RAS1 forces a valence orbital to be found at low indices: if the number of frozen orbitals is not set to zero, the contribution of this (important) orbital to the dynamic correlation would be lost.

   In particular we applied single state (SS-) RASPT2 corrections on the top of GS calculations and a multi-state (MS-) corrections on the top of the core-excited states.

6. Transition dipole moments (TDMs) computation. These are computed between the GS and the core-excited manifold of states by means of RAS state interaction (SI) routine;

7. MP2 GS normal mode and frequency calculation and numerical (MS-)RASPT2 gradients on the excited states of interest (at the Franck-Codon geometry). These are the necessary quantities to compute the vibronic couplings in the DHO model.

A similar protocol has been used to simulate XPS. In this case, however, instead of core-excited states for a neutral molecule (considered above), the ground-state of the core cation molecule has to be considered. This is achieved by decreasing the number of active electrons by 1, and by enforcing the spin multiplicity of the final state to be equal to 2. The core-hole is still produced by the application of HEXS keyword to RAS1.

In order to correctly describe core excitation and core ionization processes we employ a relativistic atomic natural orbital basis set (ANO-RCC). Table S1 reports the basis sets used to describe the atoms present in the studied molecules.

Table S1: Summary of the basis sets used for the computation of XANES and XPS spectra.

Element	Basis set
H	8s4p3d1f.2s1p
C	14s9p4d3f2g.4s3p2d
N	14s9p4d3f2g.4s3p2d1f
O	14s9p4d3f2g.4s3p2d1f
F	14s9p4d3f2g.4s3p2d1f

In particular, we employ a Double-ζ basis set for hydrogen, an augmented double-ζ for carbon, a triple-ζ for nitrogen, oxygen and fluorine.

S2.1 Level of theory of the computations

In the following we report the level of theory employed in each computation. The details of the RASSCF/RASPT2 computations are presented here in a contracted form, given as SA-a-RAS$(b, c|d, e|f, g)$/SS(or MS)PT2, where: a is the number of excited state taken into account in the state-average procedure; b is the maximum number of holes in RAS1; c is the number of orbitals in RAS1; d is the number of electron in RAS2 ; e is the number of orbitals in RAS2; f is the maximum number of electron in RAS3; g is the number of orbitals

in RAS3. The data are reported in Table S2, indicating also the spectroscopic observable that was obtained with each given computation.

Molecule	K-edge	Target state(s)	Level of Theory	Used for
Ethene	C_{H_2}	GS	RAS(1,1|2,1|4,11)/SSPT2	XANES/IP
		Core (sym. a')	SA-8-RAS(1,1|2,1|4,11)/MSPT2	XANES
		Core (sym. a'')	SA-8-RAS(1,1|2,1|4,11)MSPT2	XANES
		Core cation	RAS(1,1|2,1|2,1)/SSPT2	IP
Vinyl fluoride	C_{H_2}	GS	RAS(1,1|4,2|4,11)/SSPT2	XANES/IP
		Core (sym. a')	SA-6-RAS(1,1|4,2|4,11)/MSPT2	XANES
		Core (sym. a'')	SA-6-RAS(1,1|4,2|4,11)/MSPT2	XANES
		Core cation	RAS(1,1|4,2|2,1)/SSPT2	IP
	C_{HF}	GS	RAS(1,1|6,3|4,11)/SSPT2	XANES/IP
		Core (sym. a')	SA-6-RAS(1,1|4,2|4,11)/MSPT2	XANES
		Core (sym. a'')	SA-6-RAS(1,1|4,2|4,11)/MSPT2	XANES
		Core cation	RAS(1,1|4,2|2,1)/SSPT2	IP
	F	GS	RAS(1,1|4,2|4,11)/SSPT2	XANES/IP
		Core (sym. a')	SA-6-RAS(1,1|4,2|4,11)/MSPT2	XANES
		Core (sym. a'')	SA-6-RAS(1,1|4,2|4,11)/MSPT2	XANES
		Core cation	RAS(1,1|4,2|4,11)/SSPT2	IP
1,1-difluoroethene	C_{H_2}	GS	RAS(1,1|6,3|4,12)/SSPT2	XANES/IP
		Core (sym. a')	SA-8-RAS(1,1|6,3|4,12)/MSPT2	XANES
		Core (sym. a'')	SA-8-RAS(1,1|6,3|4,12)/MSPT2	XANES
		GS	RAS(1,1|6,3|2,1)/SSPT2	IP
		Core cation	SA-1-RAS(1,1|6,3|2,1)/SSPT2	IP
	C_{F_2}	GS	RAS(1,1|6,3|4,12)/SSPT2	XANES
		Core (sym. a')	SA-8-RAS(1,1|6,3|4,12)/MSPT2	XANES
		Core (sym. a'')	SA-8-RAS(1,1|6,3|4,12)/MSPT2	XANES

		GS	RAS(1,1|6,3|2,1)/SSPT2	IP
		Core cation	RAS(1,1|6,3|2,1)/SSPT2	IP
	F	GS	RAS(1,1|6,3|4,12)/SSPT2	IP
		Core cation	RAS(1,1|6,3|4,12)/SSPT2	IP
cis 1,2-difluoro-ethene	C_{HF}	GS	RAS(1,1|6,3|4,11)/SSPT2	XANES/IP
		Core (sym. a')	SA-8-RAS(1,1|6,3|4,11)/MSPT2	XANES
		Core (sym. a'')	SA-8-RAS(1,1|6,3|4,11)/MSPT2	XANES
		Core cation	SA-1-RAS(1,1|6,3|4,11)/SSPT2	IP
	F	GS	RAS(1,1|6,3|4,11)/SSPT2	IP
		Core cation	SA-1-RAS(1,1|6,3|4,11)/SSPT2	IP
Tetrafluoroethene	C_{F_2}	GS	RAS(1,1|10,5|4,16)/SSPT2	XANES
		Core (sym. a')	SA-6-RAS(1,1|10,5|4,16)/MSPT2	XANES
		Core (sym. a'')	SA-6-RAS(1,1|10,5|4,16)/MSPT2	XANES
Glycine (linear)	N_{H_2}	GS	RAS(1,1|12,6|4,8)/SSPT2	XANES
		Core (sym. a')	SA-4-RAS(1,1|12,6|4,8)/MSPT2	XANES
		GS	RAS(1,1|12,6|4,9)/SSPT2	XANES
		Core (sym. a'')	SA-4-RAS(1,1|12,6|4,9)/MSPT2	XANES
		GS	RAS(1,1|12,6|4,13)/SSPT2	XPS
		Core cation	RAS(1,1|12,6|4,13)/SSPT2	XPS
	O_H	GS	RAS(1,1|12,6|4,9)/SSPT2	XANES
		Core (sym. a')	SA-4-RAS(1,1|12,6|4,9)/MSPT2	XANES
		Core (sym. a'')	SA-4-RAS(1,1|12,6|4,9)/MSPT2	XANES
		GS	RAS(1,1|12,6|4,13)/SSPT2	XPS
		Core cation	RAS(1,1|12,6|4,13)/SSPT2	XPS
	O_C	GS	RAS(1,1|12,6|4,12)/SSPT2	XANES
		Core (sym. a')	SA-6-RAS(1,1|12,6|4,12)/MSPT2	XANES
		Core (sym. a'')	SA-6-RAS(1,1|12,6|4,12)/MSPT2	XANES

		GS	RAS(1,1|12,6|4,13)/SSPT2	XPS
		Core cation	SA-1-RAS(1,1|12,6|4,13)/SSPT2	XPS
	C_O	GS	RAS(1,1|12,6|4,13)/SSPT2	XPS
		Core cation	SA-1-RAS(1,1|12,6|4,13)/SSPT2	XPS
	C_{H_2}	GS	RAS(1,1|12,6|4,13)/SSPT2	XPS
		Core cation	RAS(1,1|12,6|4,13)/SSPT2	XPS
Glycine (cyclic)	N_{H_2}	GS	RAS(1,1|12,6|4,9)/SSPT2	XANES
		Core (sym. a')	SA-6-RAS(1,1|12,6|4,9)/MSPT2	XANES
		GS	RAS(1,1|12,6|4,8)/SSPT2	XANES
		Core (sym. a'')	SA-4-RAS(1,1|12,6|4,8)/MSPT2	XANES
Formaldehyde	C	GS	RAS(1,1|2,2|4,12)/SSPT2	XANES/IP
		Core (sym. a'')	SA-8-RAS(1,1|2,2|4,12)/MSPT2	XANES
		Core cation	RAS(1,1|2,2|4,12)/SSPT2	IP

Table S2: Summary of the level of theory used for the computation of XANES, XPS and IPs for the systems studied in this work. For each system, we report the excited/ionized atom, the nature of the target state (core-excited state of different symmetry or core-cation state), the level of theory employed, and the spectroscopic observable in which the calculation was used.

For the simulation of the vibronic band of formaldehyde carbon K-edge. Duschinsky and Herzberg-Teller (HT) effects are also taken into account. These can be computed providing some additional computations to be performed, namely: (a) Geometry optimization in the $1s^{[1]}\pi^{*[1]}$ state; (b) frequency and normal modes of the $1s^{[1]}\pi^{*[1]}$ state at the geometry obtained in (a); (c) Transition dipole moments derivatives in Cartesian coordinates. All these QC data are than processed by the code FC-classes which eventually produce the desired spectra.

The level of theory used to compute the above mention additional QC computations are reported in the Table below.

Table S3: Level of theory used to calculate the different QC data required for the consideration of Duschinsky and HT effects in the carbon K-edge of formaldehyde.

Targhet	Sym.	Level of theory
ES OPT.	C_{2v}	SA-4-RAS(1,1|2,2|4,14)/MSPT2
ES FREQ.	C_1	SA-8-RAS(1,1|2,2|4,12)/MSPT2
TDM deriv.	C_1	SA-8-RAS(1,1|2,2|4,12)/MSPT2

S3 Core-excited/ionized states lifetime

Here we report the phenomenological lifetimes that we employed in the computations. These give a symmetric (Lorentzian-like) contribution to the line-shape broadening of XANES and XPS spectra.

Molecule	K-edge	Lifetimes (fs)
ethene	C	1.0
Vinyl fluoride	C	1.0
	F	1.0
1,1-difluoroethene	C	1.0
cis 1,2-difluoroethene	C	1.0
tetrafluoroethene	C	1.0
glycine	C	1.25
	N	1.25
	O	1.25
Formaldehyde	C	3.3

Table S4: Core excited states lifetimes used to compute XANES and XPS for the systems studied in this work.

S4 Active spaces and multi-configurational character of core-excited states

In what follows, we present the active spaces (AS) and the main configuration state functions (CFSs) that contribute to the wave-functions of the computed core-excited states. Since all the computation are performed employing the C_s symmetry, the core-excited states belonging to different irreducible representations can be labeled accordingly. Moreover, these were computed separately, providing a speed-up of the calculations.

S4.1 Ethene

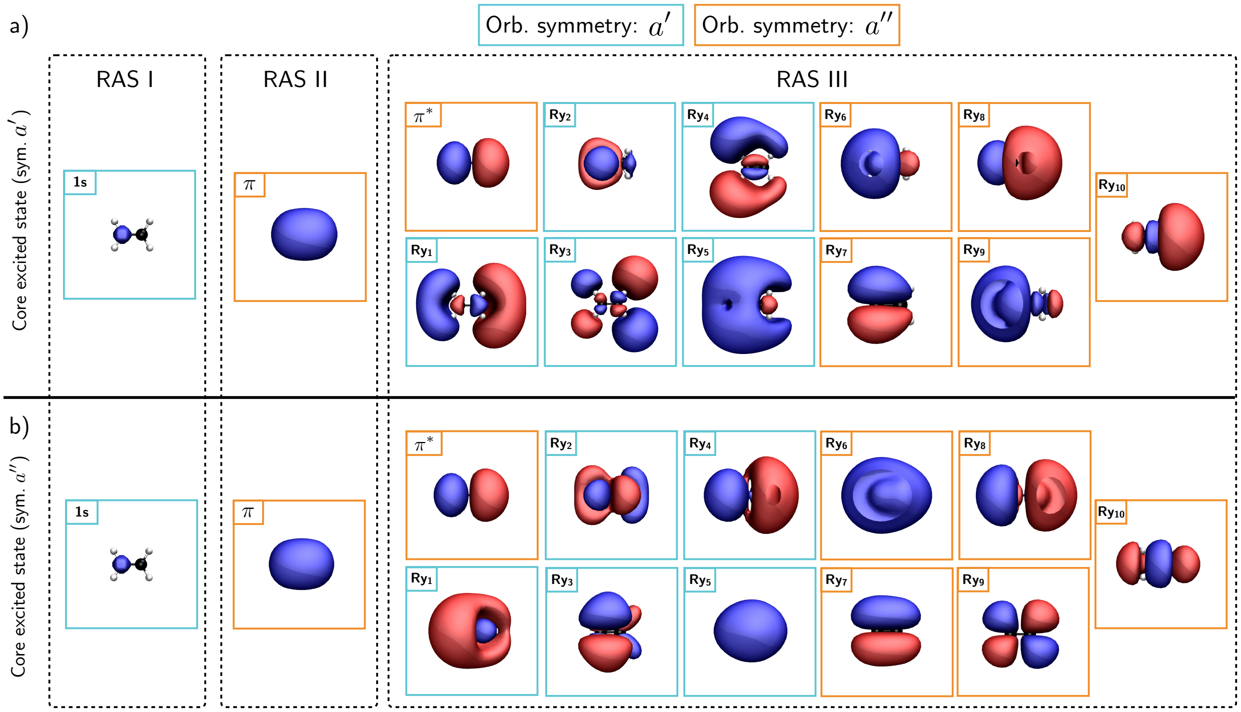

Figure S1: Active space used to compute ethene carbon K-edge XANES. a) optimized AS for states of a' symmetry. b) optimized AS for states of a'' symmetry.

Table S5: Main core excited states of a' symmetry that give rise to ethene XANES. The orbital involved in the excitation process are highlighted in figure S1-(a).

Root	ΔE	$\|TDM\|^2$	Configuration	Weight
1	287.38	5.17e-04	$1s^{[1]}Ry_5{}^{[1]}$	0.83
			$1s^{[1]}\pi^{[1]}Ry_5{}^{[1]}\pi^{*[1]}$	0.07
			$1s^{[1]}\pi^{[1]}Ry_5{}^{[1]}\pi^{*[1]}$	0.06
2	288.30	1.94e-03	$1s^{[1]}Ry_4{}^{[1]}$	0.80
			$1s^{[1]}\pi^{[1]}Ry_4{}^{[1]}\pi^{*[1]}$	0.06
			$1s^{[1]}\pi^{[1]}Ry_4{}^{[1]}\pi^{*[1]}$	0.05
3	288.46	9.18e-05	$1s^{[1]}Ry_1{}^{[1]}$	0.81
			$1s^{[1]}\pi^{[1]}Ry_1{}^{[1]}\pi^{*[1]}$	0.07
			$1s^{[1]}\pi^{[1]}Ry_1{}^{[1]}\pi^{*[1]}$	0.08
4	291.08	1.39e-03	$1s^{[1]}Ry_3{}^{[1]}$	0.76
			$1s^{[1]}\pi^{[1]}Ry_3{}^{[1]}\pi^{*[1]}$	0.08
			$1s^{[1]}\pi^{[1]}Ry_3{}^{[1]}\pi^{*[1]}$	0.08

Table S6: Main core excited states of a'' symmetry that give rise to ethene XANES. The orbital involved in the excitation process are highlighted in figure S1-(b). The shake-up state highlighted with a black arrow in Figure 3 of the main text, is here marked in red.

Root	ΔE	$\|TDM\|^2$	Configuration	Weight
1	284.96	6.10e-03	$1s^{[1]}\pi^{*[1]}$	0.81
			$1s^{[1]}\pi^{[1]}\pi^{*[2]}$	0.15
2	293.13	1.56e-03	$1s^{[1]}\pi^{*[1]}$	0.15
			$1s^{[1]}Ry_6{}^{[1]}$	0.06
			$1s^{[1]}\pi^{[1]}\pi^{*[2]}$	0.72
3	294.20	1.64e-03	$1s^{[1]}Ry_6{}^{[1]}$	0.73
			$1s^{[1]}\pi^{[1]}\pi^{*[2]}$	0.09
			$1s^{[1]}\pi^{[1]}\pi^{*[1]}Ry_6{}^{[1]}$	0.05
			$1s^{[1]}\pi^{[1]}\pi^{*[1]}Ry_6{}^{[1]}$	0.06
5	297.36	8.53e-10	$1s^{[1]}Ry_8{}^{[1]}$	0.72
			$1s^{[1]}\pi^{[1]}\pi^{*[1]}Ry_8{}^{[1]}$	0.05
			$1s^{[1]}\pi^{[1]}\pi^{*[1]}Ry_8{}^{[1]}$	0.08

S4.2 Vinyl fluoride

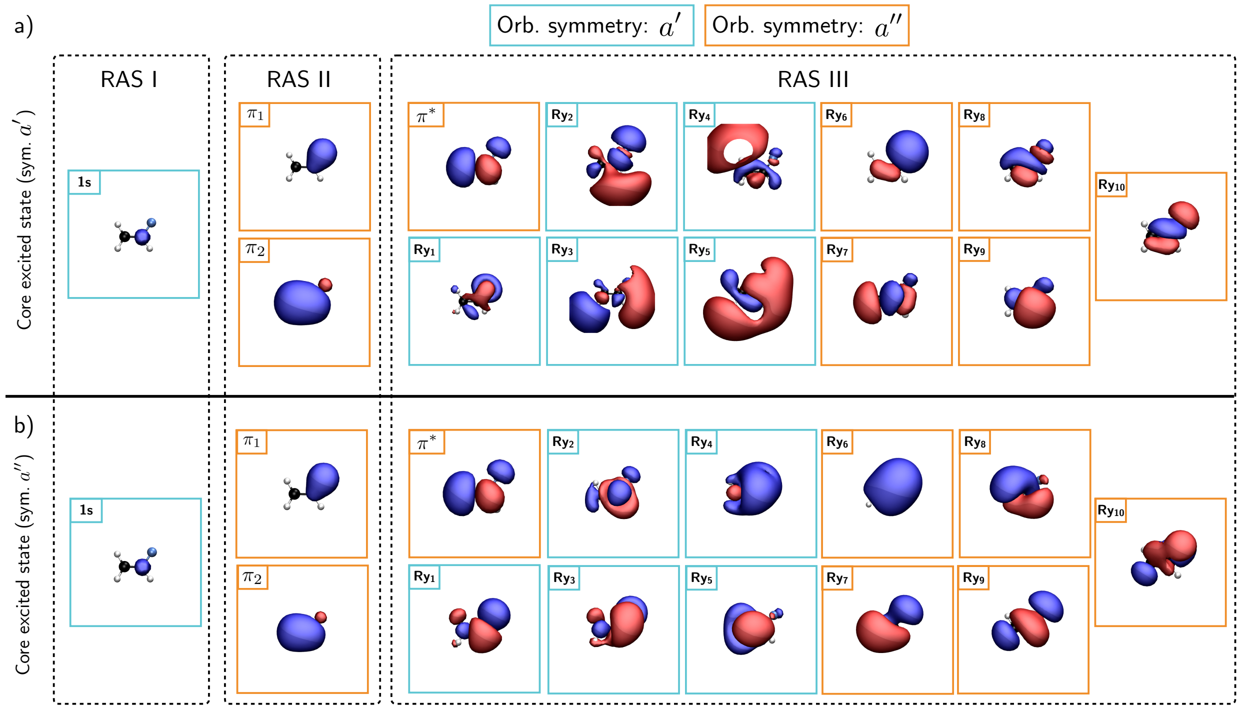

Figure S2: Active space used to compute C_{HF} K-edge XANES of vinyl fluoride. a) optimized AS for states of a' symmetry. b) optimized AS for states of a'' symmetry.

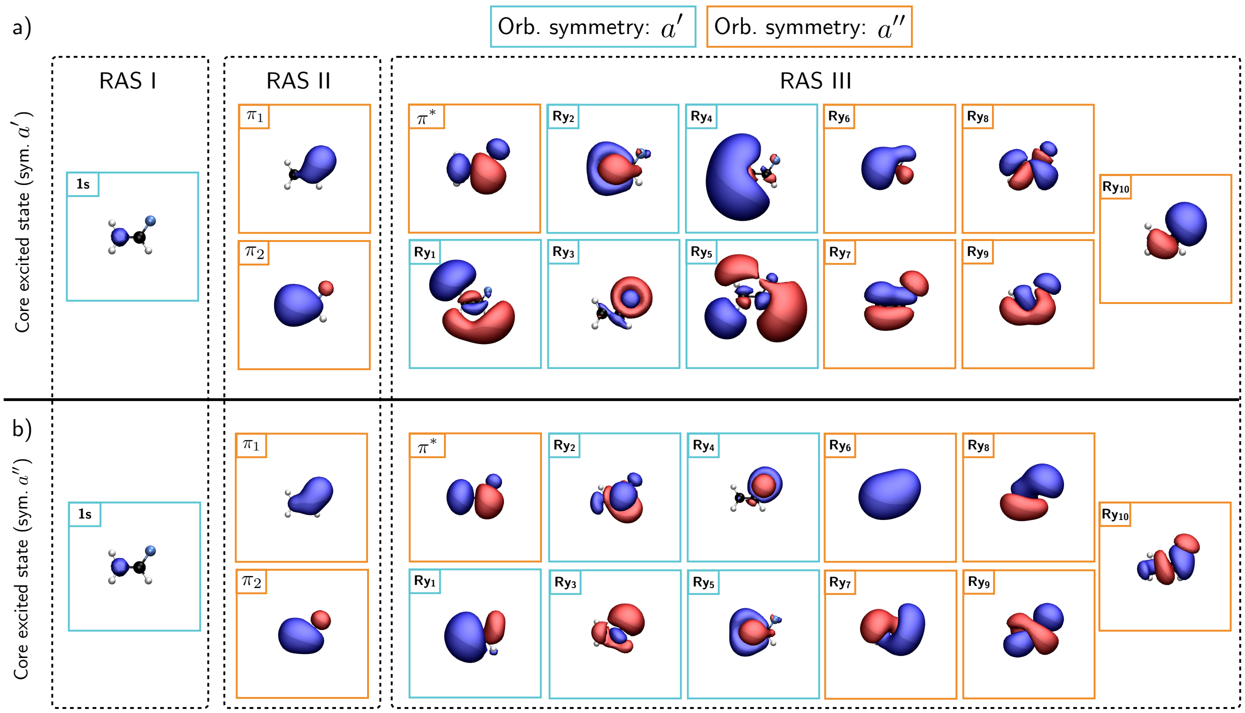

Figure S3: Active space used to compute C_{H_2} K-edge XANES of vinyl fluoride. a) optimized AS for states of a' symmetry. b) optimized AS for states of a'' symmetry.

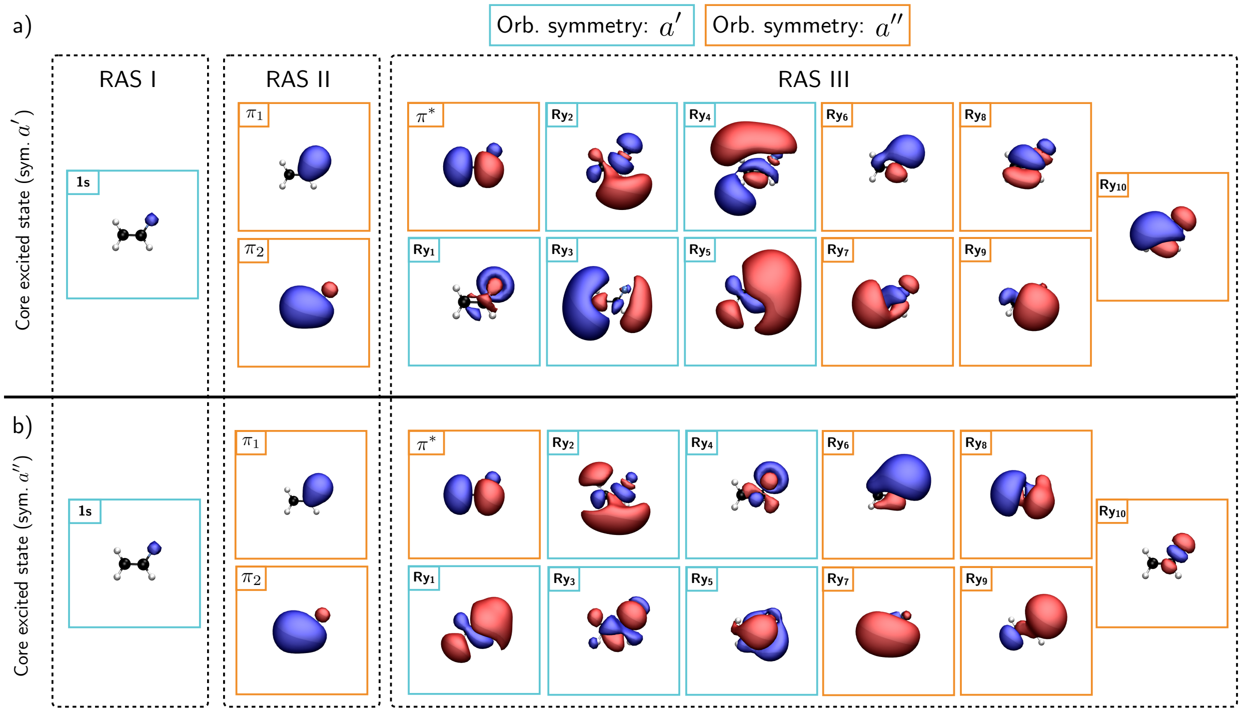

Figure S4: Active space used to compute F K-edge XANES of Vinyl fluoride. a) optimized AS for states of a' symmetry. b) optimized AS for states of a'' symmetry.

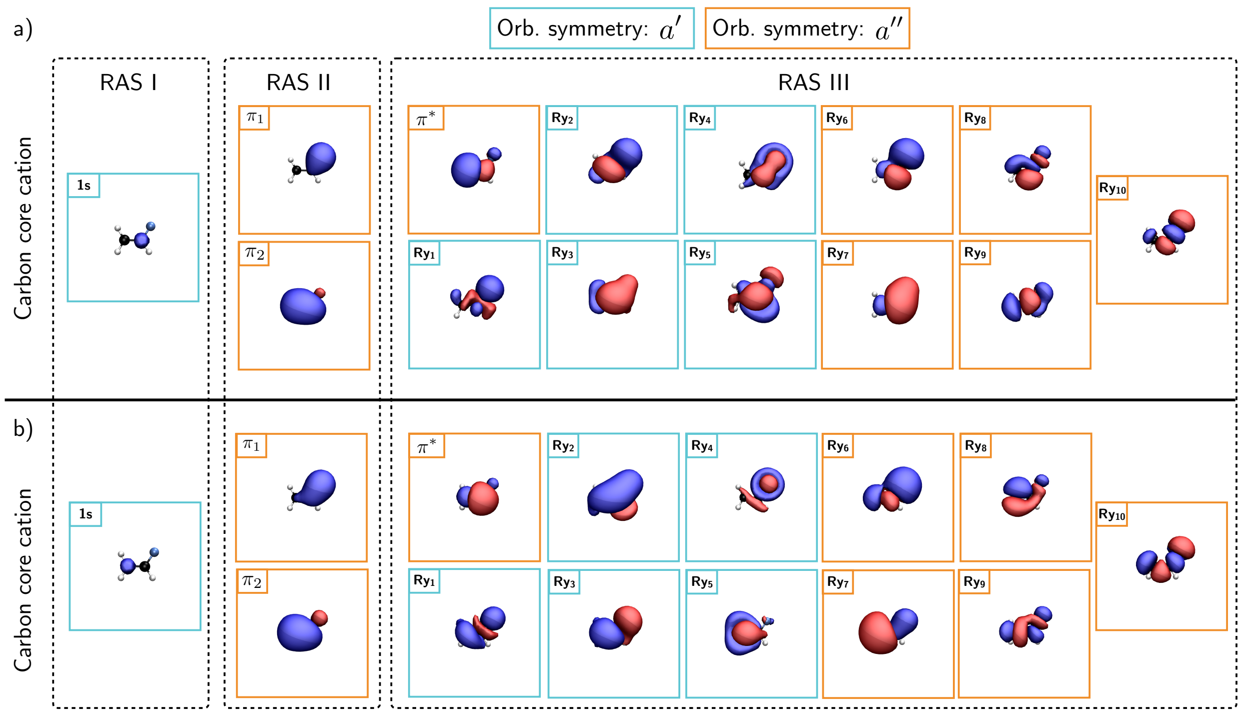

Figure S5: Active space used to compute the ionization potentials for the two carbon atoms of Vinyl fluoride. a) optimized AS for C_{HF}. b) optimized AS for C_{H_2}.

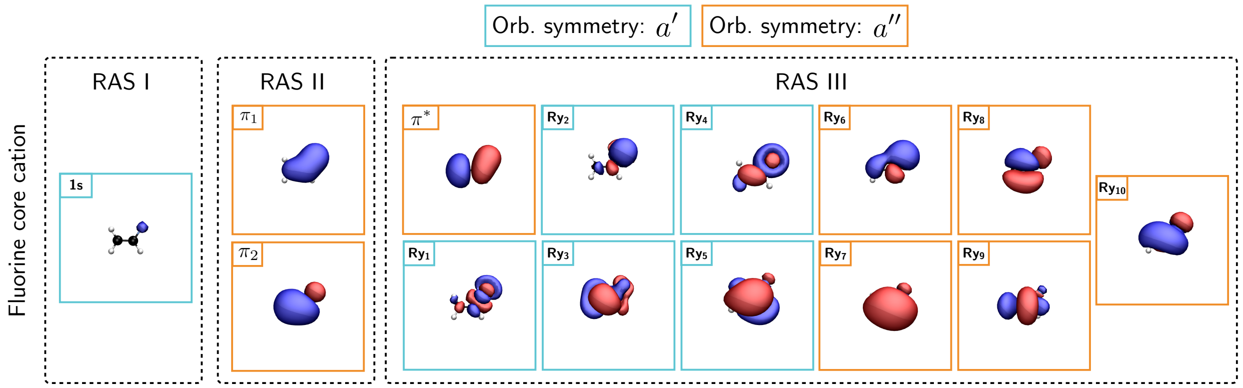

Figure S6: Active space used to compute the F ionization potentials for Vinyl fluoride.

Table S7: Core excited states of a' symmetry that give rise to the carbon K-edge XANES of Vinyl fluoride. The orbital involved in the excitation process are highlighted in figure S2-(a).

Root	ΔE	$\|TDM\|^2$	Configuration	Weight
1	289.93	2.23e-03	$1s^{[1]} Ry_5{}^{[1]}$	0.85
2	290.79	1.92e-03	$1s^{[1]} Ry_2{}^{[1]}$	0.20
			$1s^{[1]} Ry_3{}^{[1]}$	0.19
			$1s^{[1]} Ry_4{}^{[1]}$	0.50
3	291.11	2.99e-03	$1s^{[1]} Ry_2{}^{[1]}$	0.62
			$1s^{[1]} Ry_4{}^{[1]}$	0.25

Table S8: Core excited states of a'' symmetry that give rise to the carbon K-edge XANES of Vinyl fluoride. The orbital involved in the excitation process are highlighted in figure S2-(b). The shake-up state highlighted with a black arrow in Figure 3 of the main text, is here marked in red.

Root	ΔE	$\|TDM\|^2$	Configuration	Weight
1	287.21	6.72e-03	$1s^{[1]} \pi^{*[1]}$	0.74
			$1s^{[1]} \pi_2{}^{[1]} \pi^{*[2]}$	0.14
2	294.60	1.59e-03	$1s^{[1]} \pi^{*[1]}$	0.10
			$1s^{[1]} Ry_6{}^{[1]}$	0.08
			$1s^{[1]} \pi_2{}^{[1]} \pi^{*[2]}$	0.67
3	295.63	6.64e-04	$1s^{[1]} Ry_6{}^{[1]}$	0.64
			$1s^{[1]} \pi_2{}^{[1]} \pi^{*[2]}$	0.07
			$1s^{[1]} \pi_2{}^{[1]} \pi^{*[1]} Ry_6{}^{[1]}$	0.07
4	298.62	1.30e-04	$1s^{[1]} Ry_7{}^{[1]}$	0.60
			$1s^{[1]} Ry_8{}^{[1]}$	0.08
			$1s^{[1]} \pi_2{}^{[1]} \pi^{*[1]} Ry_7{}^{[1]}$	0.08
			$1s^{[1]} \pi_2{}^{[1]} \pi^{*[1]} Ry_7{}^{[1]}$	0.05
5	300.05	1.08e-03	$1s^{[1]} Ry_8{}^{[1]}$	0.61
			$1s^{[1]} \pi_2{}^{[1]} \pi^{*[1]} Ry_8{}^{[1]}$	0.08
			$1s^{[1]} \pi_2{}^{[1]} \pi^{*[1]} Ry_8{}^{[1]}$	0.07
6	301.24	1.21e-04	$1s^{[1]} \pi_2{}^{[1]} \pi^{*[1]} Ry_6{}^{[1]}$	0.59
			$1s^{[1]} \pi_2{}^{[1]} \pi^{*[1]} Ry_6{}^{[1]}$	0.23

Table S9: Core excited states of a' symmetry that give rise to the carbon K-edge XANES of Vinyl fluoride. The orbital involved in the excitation process are highlighted in figure S3-(a).

Root	ΔE	$\|TDM\|^2$	Configuration	Weight
1	287.41	6.97e-04	$1s^{[1]}Ry_1{}^{[1]}$	0.06
			$1s^{[1]}Ry_4{}^{[1]}$	0.69
			$1s^{[1]}\pi_2{}^{[1]}Ry_4{}^{[1]}\pi^{*[1]}$	0.15
2	288.21	9.12e-04	$1s^{[1]}Ry_1{}^{[1]}$	0.73
			$1s^{[1]}Ry_4{}^{[1]}$	0.05
			$1s^{[1]}\pi_2{}^{[1]}Ry_1{}^{[1]}\pi^{*[1]}$	0.13
3	289.34	1.65e-03	$1s^{[1]}Ry_5{}^{[1]}$	0.77
			$1s^{[1]}\pi_2{}^{[1]}Ry_5{}^{[1]}\pi^{*[1]}$	0.13

Table S10: Core excited states of a'' symmetry that give rise to the carbon K-edge XANES of Vinyl fluoride. The orbital involved in the excitation process are highlighted in figure S3-(b). The shake-up state highlighted with a black arrow in Figure 3 of the main text, is here marked in red.

Root	ΔE	$\|TDM\|^2$	Configuration	Weight
1	285.27	5.71e-03	$1s^{[1]}\pi^{*[1]}$	0.82
			$1s^{[1]}\pi_2{}^{[1]}\pi^{*[2]}$	0.13
2	293.32	1.97e-03	$1s^{[1]}Ry_6{}^{[1]}$	0.72
			$1s^{[1]}\pi_2{}^{[1]}\pi^{*[1]}Ry_6{}^{[1]}$	0.05
3	294.30	3.04e-04	$1s^{[1]}\pi^{*[1]}$	0.10
			$1s^{[1]}\pi_2{}^{[1]}\pi^{*[2]}$	0.77
4	296.28	4.48e-04	$1s^{[1]}Ry_8{}^{[1]}$	0.24
			$1s^{[1]}\pi_1{}^{[1]}\pi^{*[2]}$	0.56
5	296.43	7.57e-04	$1s^{[1]}Ry_8{}^{[1]}$	0.55
			$1s^{[1]}\pi_1{}^{[1]}\pi^{*[2]}$	0.31
6	297.80	3.19e-03	$1s^{[1]}Ry_6{}^{[1]}$	0.06
			$1s^{[1]}Ry_7{}^{[1]}$	0.72
			$1s^{[1]}\pi_2{}^{[1]}\pi^{*[1]}Ry_6{}^{[1]}$	0.05
			$1s^{[1]}\pi_2{}^{[1]}\pi^{*[1]}Ry_7{}^{[1]}$	0.05

Table S11: Core excited states of a' symmetry that give rise to the fluorine K-edge XANES of Vinyl fluoride. The orbital involved in the excitation process are highlighted in figure S4-(a).

Root	ΔE	$\|TDM\|^2$	Configuration	Weight
1	690.99	3.51e-04	$1s^{[1]} Ry_5{}^{[1]}$	0.81
2	691.59	7.26e-04	$1s^{[1]} Ry_2{}^{[1]}$	0.70
			$1s^{[1]} Ry_3{}^{[1]}$	0.05
			$1s^{[1]} Ry_4{}^{[1]}$	0.07

Table S12: Core excited states of a'' symmetry that give rise to the fluorine K-edge XANES of Vinyl fluoride. The orbital involved in the excitation process are highlighted in figure S4-(b).

Root	ΔE	$\|TDM\|^2$	Configuration	Weight
1	690.10	3.16e-04	$1s^{[1]} \pi^{*[1]}$	0.85
2	696.09	3.17e-04	$1s^{[1]} Ry_6{}^{[1]}$	0.58
			$1s^{[1]} Ry_7{}^{[1]}$	0.14
			$1s^{[1]} \pi_2{}^{[1]} \pi^{*[2]}$	0.12
3	696.80	7.35e-05	$1s^{[1]} Ry_6{}^{[1]}$	0.09
			$1s^{[1]} \pi_2{}^{[1]} \pi^{*[2]}$	0.70
6	699.85	2.15e-04	$1s^{[1]} Ry_6{}^{[1]}$	0.14
			$1s^{[1]} Ry_7{}^{[1]}$	0.60

S4.3 1,1-difluoroethene

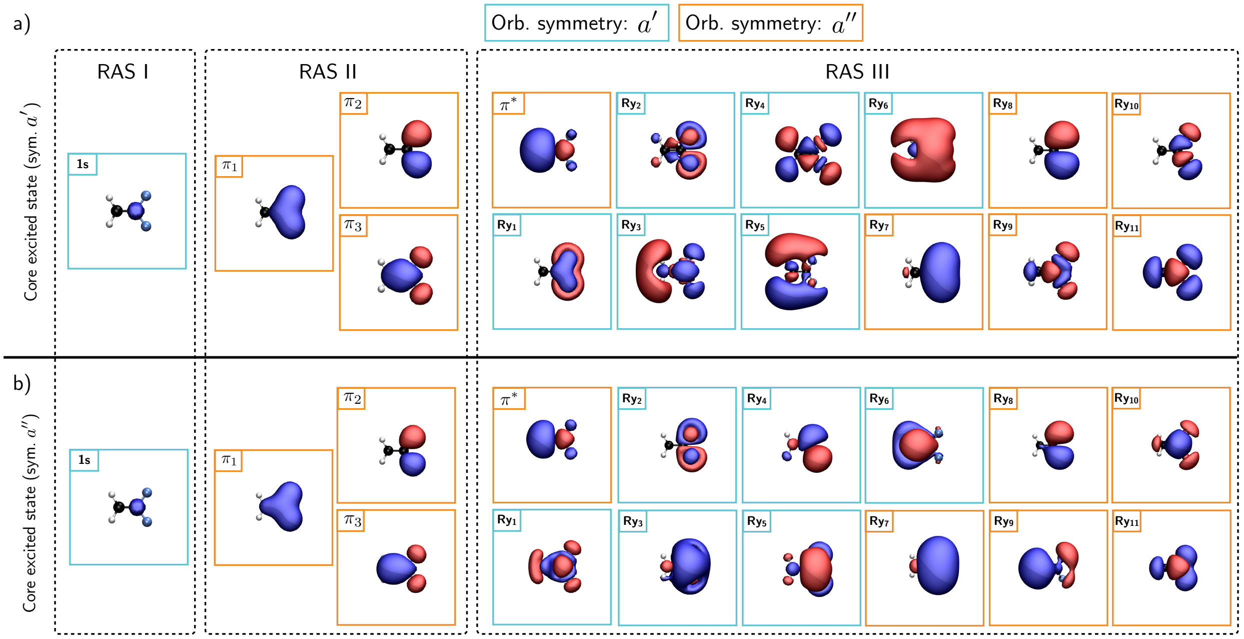

Figure S7: Active space used to compute C_{F_2} K-edge XANES of 1,1-difluoroethene. a) optimized AS for states of a' symmetry. b) optimized AS for states of a'' symmetry.

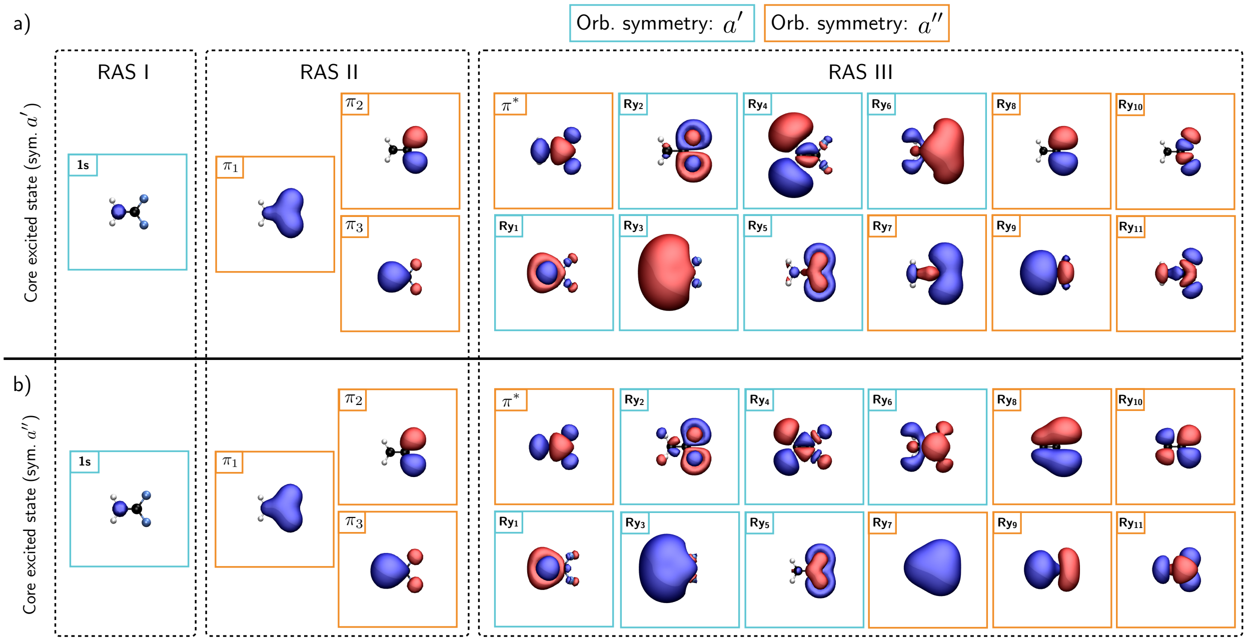

Figure S8: Active space used to compute C_{H_2} K-edge XANES of 1,1-difluoroethene. a) optimized AS for states of a' symmetry. b) optimized AS for states of a'' symmetry.

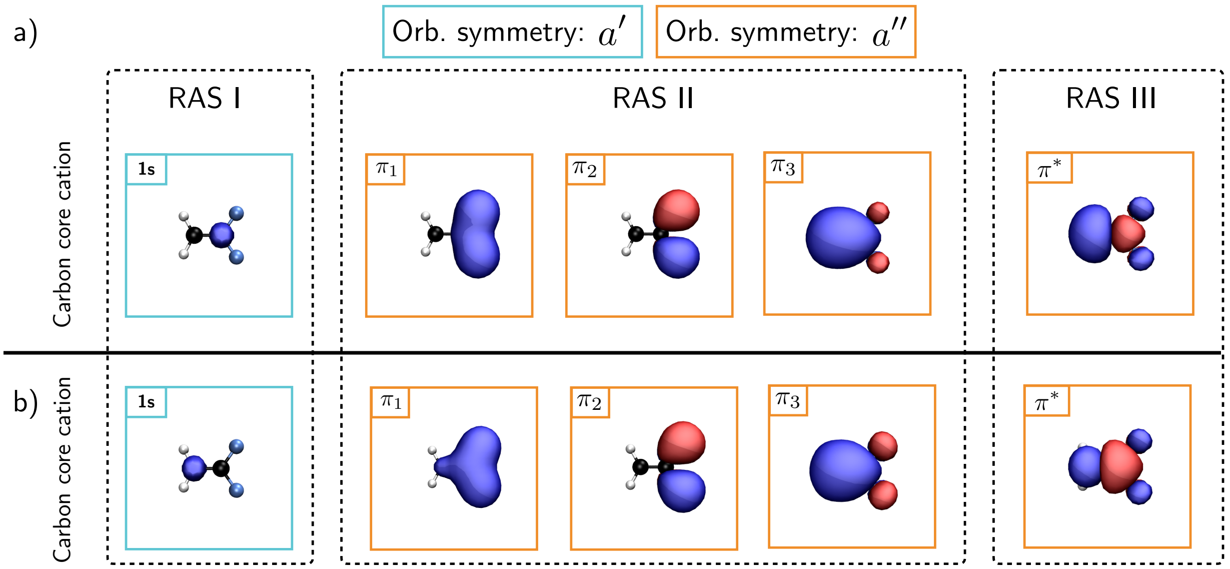

Figure S9: Active space used to compute the ionization potentials for the two carbon atoms of 1,1-difluoroethene. a) optimized AS for C_{F_2}. b) optimized AS for C_{H_2}.

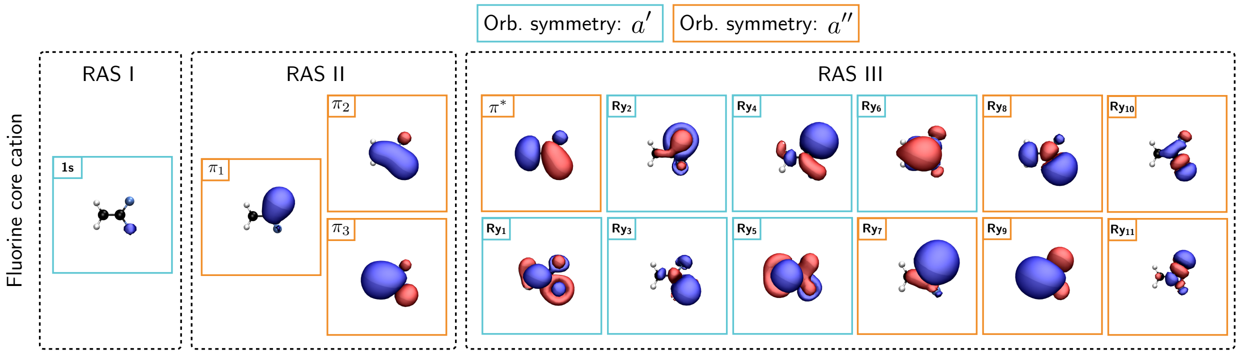

Figure S10: Active space used to compute the ionization potential for the fluorine atom of 1,1-difluoroethene.

Table S13: Core excited states of a' symmetry that give rise to the carbon K-edge XANES of 1,1-difluoroethene. The orbital involved in the excitation process are highlighted in figure S7-(a).

Root	ΔE	$\|TDM\|^2$	Configuration	Weight
1	292.42	5.98e-04	$1s^{[1]} Ry_6{}^{[1]}$	0.63
			$1s^{[1]} \pi_3{}^{[1]} Ry_6{}^{[1]} \pi^{*[1]}$	0.27
2	292.76	7.00e-03	$1s^{[1]} Ry_4{}^{[1]}$	0.53
			$1s^{[1]} Ry_5{}^{[1]}$	0.07
			$1s^{[1]} \pi_3{}^{[1]} Ry_4{}^{[1]} \pi^{*[1]}$	0.05
			$1s^{[1]} \pi_3{}^{[1]} Ry_4{}^{[1]} \pi^{*[1]}$	0.32
3	293.73	2.43e-03	$1s^{[1]} Ry_3{}^{[1]}$	0.60
			$1s^{[1]} \pi_3{}^{[1]} Ry_3{}^{[1]} \pi^{*[1]}$	0.06
			$1s^{[1]} \pi_3{}^{[1]} Ry_3{}^{[1]} \pi^{*[1]}$	0.26
4	294.56	2.24e-03	$1s^{[1]} Ry_5{}^{[1]}$	0.65
			$1s^{[1]} \pi_3{}^{[1]} Ry_5{}^{[1]} \pi^{*[1]}$	0.23

Table S14: Core excited states of a'' symmetry that give rise to the carbon K-edge XANES of 1,1-difluoroethene. The orbital involved in the excitation process are highlighted in figure S7-(b). The shake-up state highlighted with a black arrow in Figure 3 of the main text, is here marked in red.

Root	ΔE	$\|TDM\|^2$	Configuration	Weight
1	290.01	8.29e-03	$1s^{[1]} \pi^{*[1]}$	0.94
2	296.76	1.60e-03	$1s^{[1]} \pi_3{}^{[1]} \pi^{*[2]}$	0.90
3	297.66	2.03e-04	$1s^{[1]} Ry_7{}^{[1]}$	0.81

Table S15: Core excited states of a' symmetry that give rise to the carbon K-edge XANES of 1,1-difluoroethene. The orbital involved in the excitation process are highlighted in figure S8-(a).

Root	ΔE	$\|TDM\|^2$	Configuration	Weight
1	287.35	2.10e-04	$1s^{[1]} Ry_3{}^{[1]}$	0.79
			$1s^{[1]} \pi_3{}^{[1]} Ry_3{}^{[1]} \pi^{*[1]}$	0.12
2	288.49	3.18e-03	$1s^{[1]} Ry_4{}^{[1]}$	0.72
			$1s^{[1]} \pi_3{}^{[1]} Ry_4{}^{[1]} \pi^{*[1]}$	0.21
3	289.53	1.49e-03	$1s^{[1]} Ry_6{}^{[1]}$	0.82
			$1s^{[1]} \pi_3{}^{[1]} Ry_6{}^{[1]} \pi^{*[1]}$	0.10

Table S16: Core excited states of a'' symmetry that give rise to the carbon K-edge XANES of 1,1-difluoroethene. The orbital involved in the excitation process are highlighted in figure S8-(b). The shake-up state highlighted with a black arrow in Figure 3 of the main text, is here marked in red.

Root	ΔE	$\|TDM\|^2$	Configuration	Weight
1	285.58	4.65e-03	$1s^{[1]} \pi^{*[1]}$	0.90
2	292.80	1.30e-03	$1s^{[1]} Ry_7{}^{[1]}$	0.83
			$1s^{[1]} \pi_3{}^{[1]} \pi^{*[1]} Ry_7{}^{[1]}$	0.08
3	295.64	6.52e-04	$1s^{[1]} \pi_3{}^{[1]} \pi^{*[2]}$	0.91

S4.4 Cis 1,2-difluoroethene

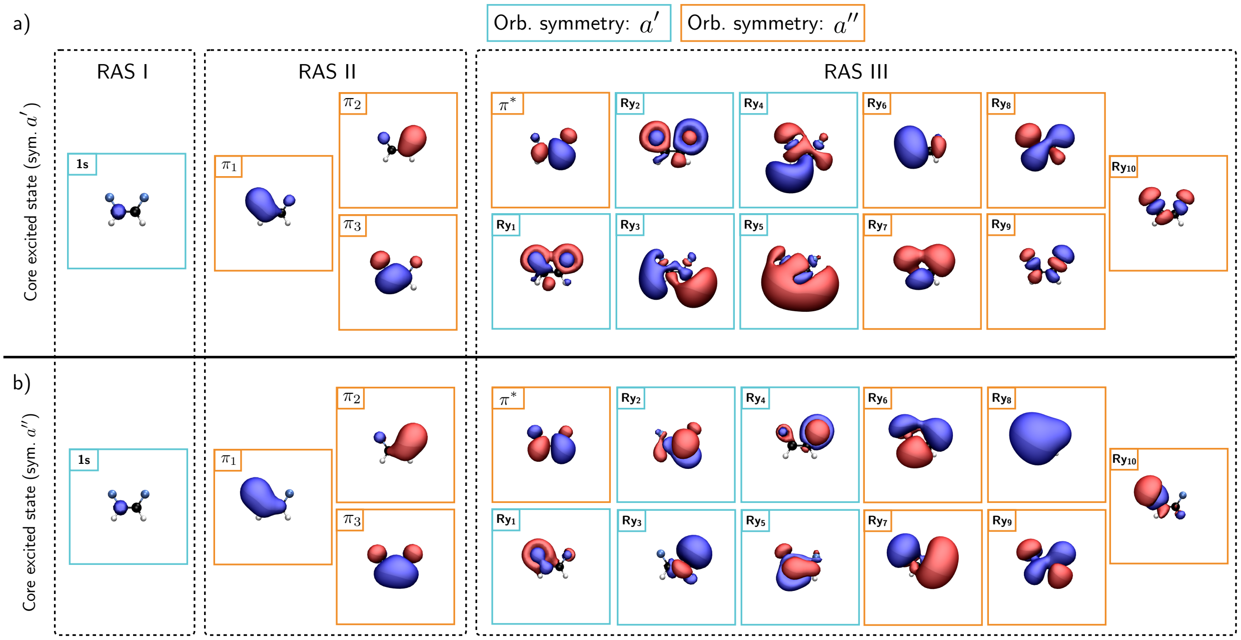

Figure S11: Active space used to compute C K-edge XANES of cis 1,2-difluoroethene. a) optimized AS for states of a' symmetry. b) optimized AS for states of a'' symmetry.

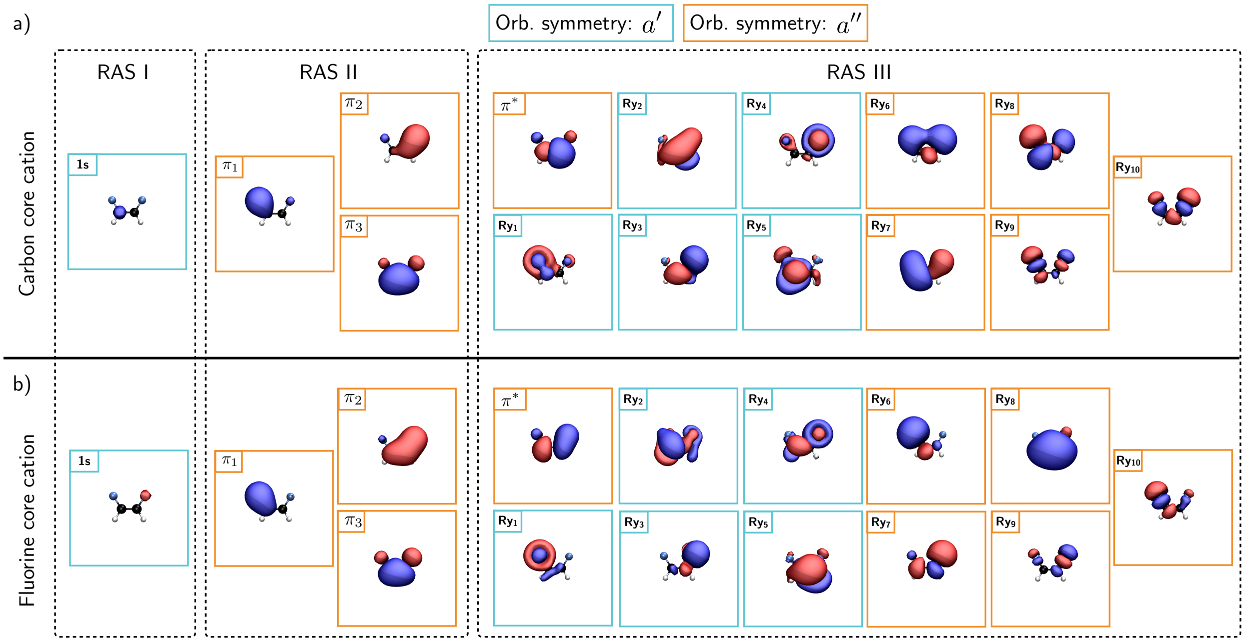

Figure S12: Active space used to compute the C and F ionization potentials for cis 1,2-difluoroethene. a) optimized AS used for the computation of C IP. b) optimized AS used for the computation of F IP.

Table S17: Core excited states of a' symmetry that give rise to the carbon K-edge XANES of cis 1,2-difluoroethene. The orbital involved in the excitation process are highlighted in figure S11-(a).

Root	ΔE	$\|TDM\|^2$	Configuration	Weight
1	289.12	3.50e-03	$1s^{[1]}Ry_5^{[1]}$	0.59
			$1s^{[1]}\pi_3^{[1]}Ry_5^{[1]}\pi^{*[1]}$	0.22
2	290.16	1.87e-03	$1s^{[1]}Ry_4^{[1]}$	0.54
			$1s^{[1]}Ry_5^{[1]}$	0.08
			$1s^{[1]}\pi_3^{[1]}Ry_4^{[1]}\pi^{*[1]}$	0.26
3	291.08	6.43e-04	$1s^{[1]}Ry_3^{[1]}$	0.67
			$1s^{[1]}\pi_3^{[1]}Ry_3^{[1]}\pi^{*[1]}$	0.19

Table S18: Core excited states of a'' symmetry that give rise to the carbon K-edge XANES of cis 1,2-difluoroethene. The orbital involved in the excitation process are highlighted in figure S11-(b). The shake-up state highlighted with a black arrow in Figure 3 of the main text, is here marked in red.

Root	ΔE	$\|TDM\|^2$	Configuration	Weight
1	287.61	6.61e-03	$1s^{[1]}\pi^{*[1]}$	0.78
			$1s^{[1]}\pi_3^{[1]}\pi^{*[2]}$	0.17
2	295.18	1.72e-03	$1s^{[1]}Ry_7^{[1]}$	0.06
			$1s^{[1]}Ry_8^{[1]}$	0.66
			$1s^{[1]}\pi_3^{[1]}\pi^{*[1]}Ry_8^{[1]}$	0.06
			$1s^{[1]}\pi_3^{[1]}\pi^{*[1]}Ry_8^{[1]}$	0.06
3	296.13	5.32e-04	$1s^{[1]}\pi^{*[1]}$	0.14
			$1s^{[1]}Ry_8^{[1]}$	0.05
			$1s^{[1]}\pi_3^{[1]}\pi^{*[2]}$	0.73
4	298.31	3.03e-04	$1s^{[1]}Ry_7^{[1]}$	0.53
			$1s^{[1]}Ry_8^{[1]}$	0.10
			$1s^{[1]}\pi_3^{[1]}\pi^{*[1]}Ry_7^{[1]}$	0.08
			$1s^{[1]}\pi_2^{[1]}\pi^{*[2]}$	0.15
			$1s^{[1]}\pi_3^{[1]}\pi^{*[1]}Ry_7^{[1]}$	0.08
5	298.87	1.96e-05	$1s^{[1]}Ry_7^{[1]}$	0.13
			$1s^{[1]}\pi_2^{[1]}\pi^{*[2]}$	0.75
6	301.20	2.60e-04	$1s^{[1]}\pi_3^{[1]}\pi^{*[1]}Ry_8^{[1]}$	0.65
			$1s^{[1]}\pi_3^{[1]}\pi^{*[1]}Ry_8^{[1]}$	0.26

S4.5 Tetrafluoroethene

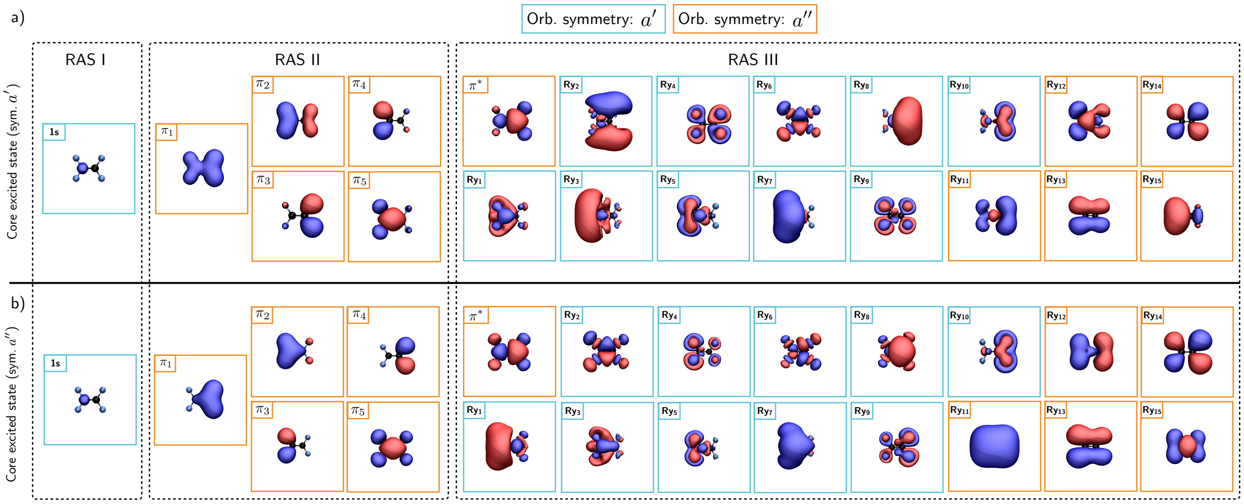

Figure S13: Active space used to compute C K-edge XANES of tetrafluoroethene. a) optimized AS for states of a' symmetry. b) optimized AS for states of a'' symmetry.

Table S19: Core excited states of a' symmetry that give rise to the carbon K-edge XANES of tetrafuoloethene. The orbital involved in the excitation process are highlighted in figure S13-(a).

Root	ΔE	$\|TDM\|^2$	Configuration	Weight
1	291.92	7.08e-03	$1s^{[1]} Ry_6{}^{[1]}$	0.64
			$1s^{[1]} \pi_5{}^{[1]} Ry_6{}^{[1]} \pi^{*[1]}$	0.26
2	292.90	2.08e-03	$1s^{[1]} Ry_7{}^{[1]}$	0.34
			$1s^{[1]} Ry_8{}^{[1]}$	0.39
			$1s^{[1]} \pi_5{}^{[1]} Ry_7{}^{[1]} \pi^{*[1]}$	0.14
3	293.63	3.93e-04	$1s^{[1]} Ry_7{}^{[1]}$	0.27
			$1s^{[1]} Ry_8{}^{[1]}$	0.44
			$1s^{[1]} \pi_5{}^{[1]} Ry_7{}^{[1]} \pi^{*[1]}$	0.06
			$1s^{[1]} \pi_5{}^{[1]} Ry_7{}^{[1]} \pi^{*[1]}$	0.12
			$1s^{[1]} \pi_5{}^{[1]} Ry_8{}^{[1]} \pi^{*[1]}$	0.06
4	296.73	2.84e-03	$1s^{[1]} Ry_2{}^{[1]}$	0.72
			$1s^{[1]} \pi_5{}^{[1]} Ry_2{}^{[1]} \pi^{*[1]}$	0.16
5	297.29	4.61e-05	$1s^{[1]} \pi_5{}^{[1]} Ry_6{}^{[1]} \pi^{*[1]}$	0.71
			$1s^{[1]} \pi_5{}^{[1]} Ry_6{}^{[1]} \pi^{*[1]}$	0.21
6	298.56	2.51e-03	$1s^{[1]} Ry_3{}^{[1]}$	0.55
			$1s^{[1]} \pi_5{}^{[1]} Ry_3{}^{[1]} \pi^{*[1]}$	0.06
			$1s^{[1]} \pi_5{}^{[1]} Ry_3{}^{[1]} \pi^{*[1]}$	0.24
			$1s^{[1]} \pi_5{}^{[1]} Ry_7{}^{[1]} \pi^{*[1]}$	0.10

Table S20: Core excited states of a'' symmetry that give rise to the carbon K-edge XANES of tetrafluoroethene. The orbital involved in the excitation process are highlighted in figure S11-(b). The The shake-up state highlighted with a black arrow in Figure 3 of the main text, is here marked in red.

Root	ΔE	$\|TDM\|^2$	Configuration	Weight
1	290.66	8.98e-03	$1s^{[1]}\pi^{*[1]}$	0.86
			$1s^{[1]}{\pi_5}^{[1]}\pi^{*[2]}$	0.07
2	297.13	7.31e-04	$1s^{[1]}{Ry_{11}}^{[1]}$	0.87
3	298.51	8.50e-04	$1s^{[1]}\pi^{*[1]}$	0.06
			$1s^{[1]}{\pi_5}^{[1]}\pi^{*[2]}$	0.78
4	299.53	1.88e-05	$1s^{[1]}{\pi_5}^{[1]}{Ry_2}^{[2]}$	0.82
			$1s^{[1]}{\pi_3}^{[1]}{Ry_2}^{[1]}{Ry_7}^{[1]}$	0.06

S4.6 Glycine

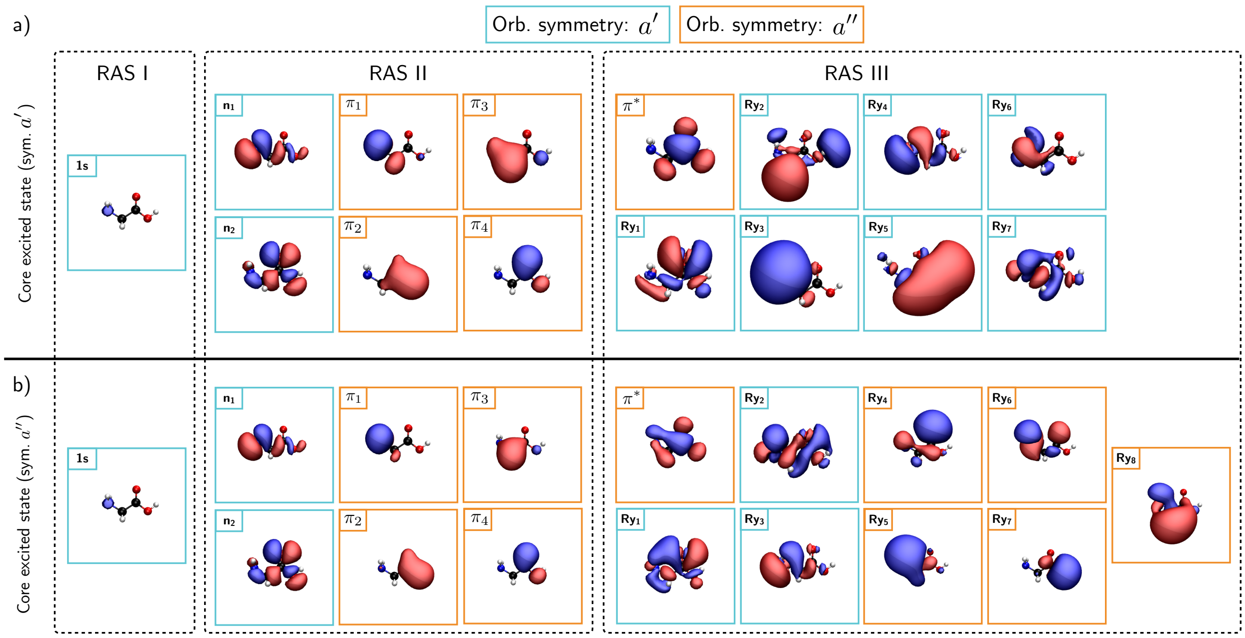

Figure S14: Active space used to compute N K-edge XANES of glycine. a) optimized AS for states of a' symmetry. b) optimized AS for states of a'' symmetry.

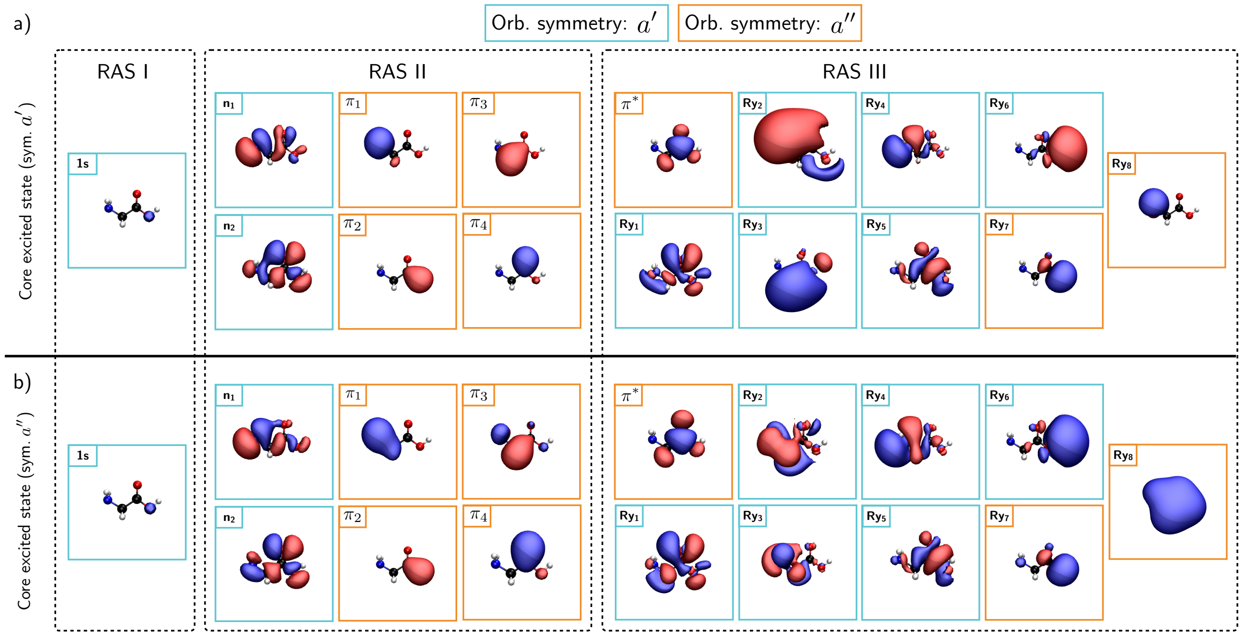

Figure S15: Active space used to compute O_H K-edge XANES of glycine. a) optimized AS for states of a' symmetry. b) optimized AS for states of a'' symmetry.

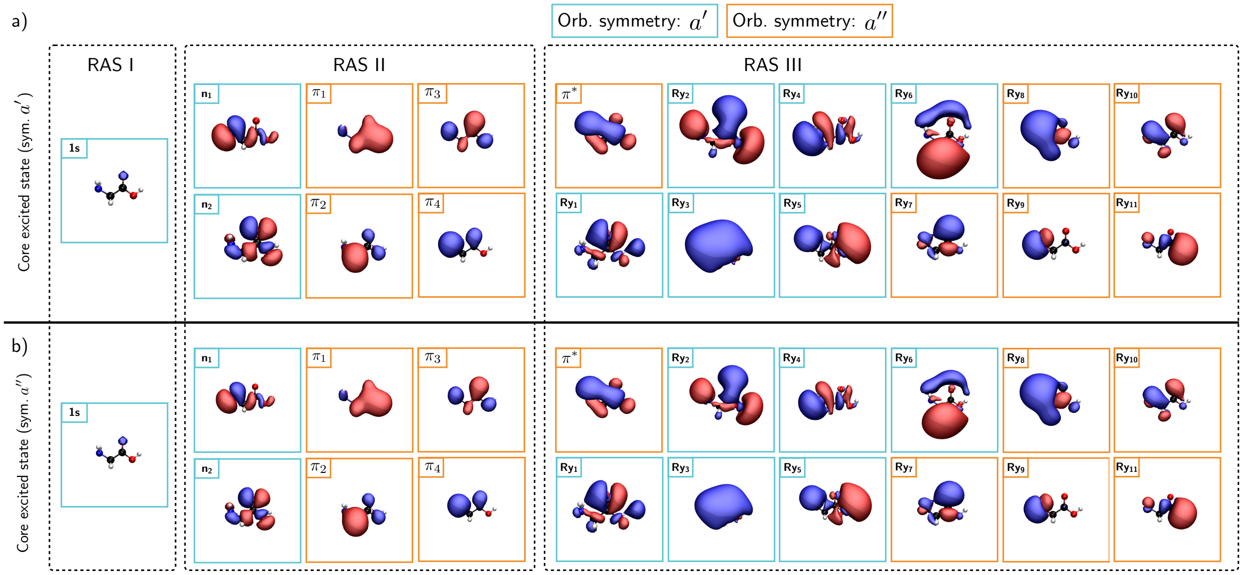

Figure S16: Active space used to compute O_C K-edge XANES of glycine. a) optimized AS for states of a' symmetry. b) optimized AS for states of a'' symmetry.

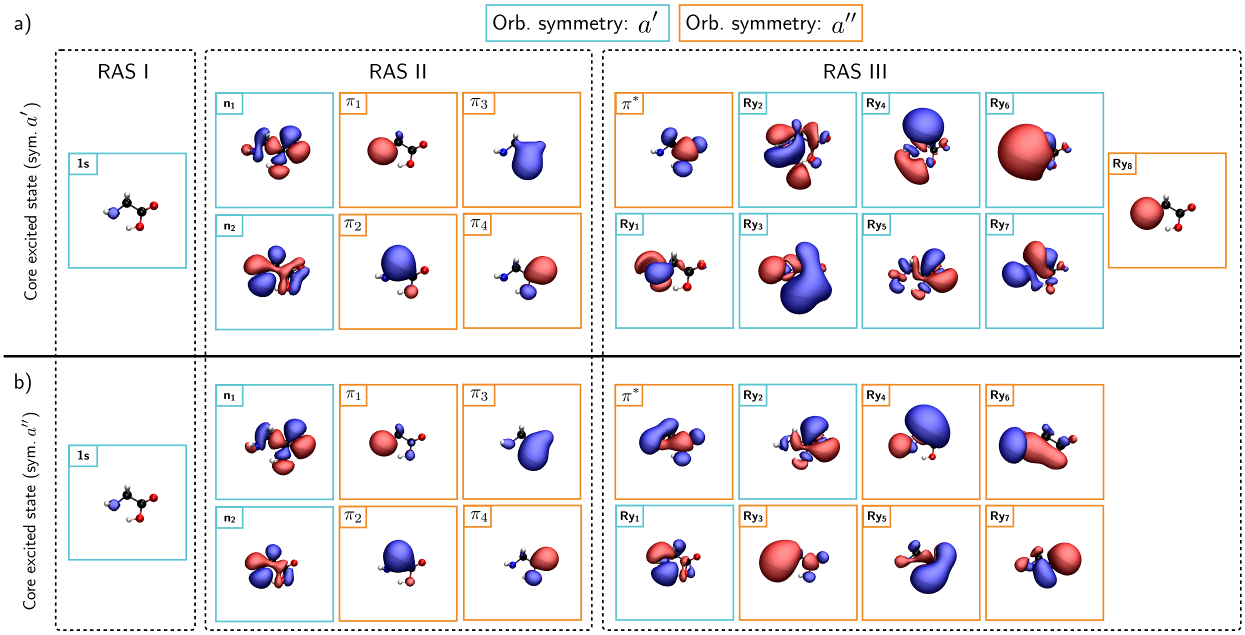

Figure S17: Active space used to compute N K-edge XANES of glycine in the "cyclyc" conformer which present intramolecular hydrogen bond. a) optimized AS for states of a' symmetry. b) optimized AS for states of a'' symmetry.

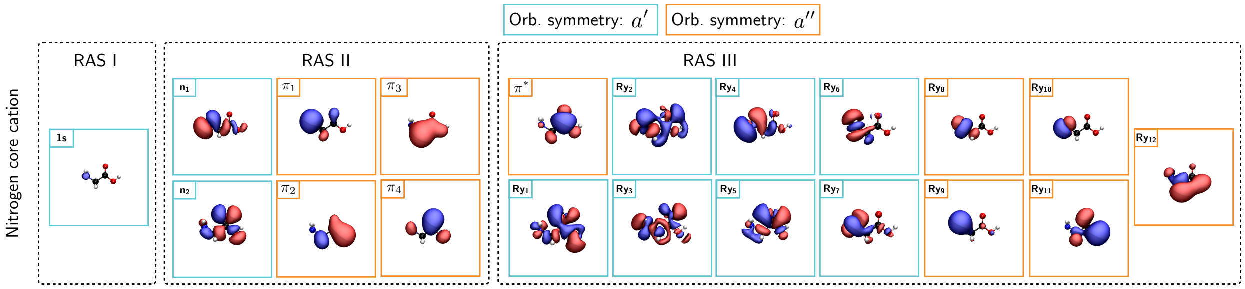

Figure S18: Active space used to compute N K-edge XPS of glycine in the "linear" conformation.

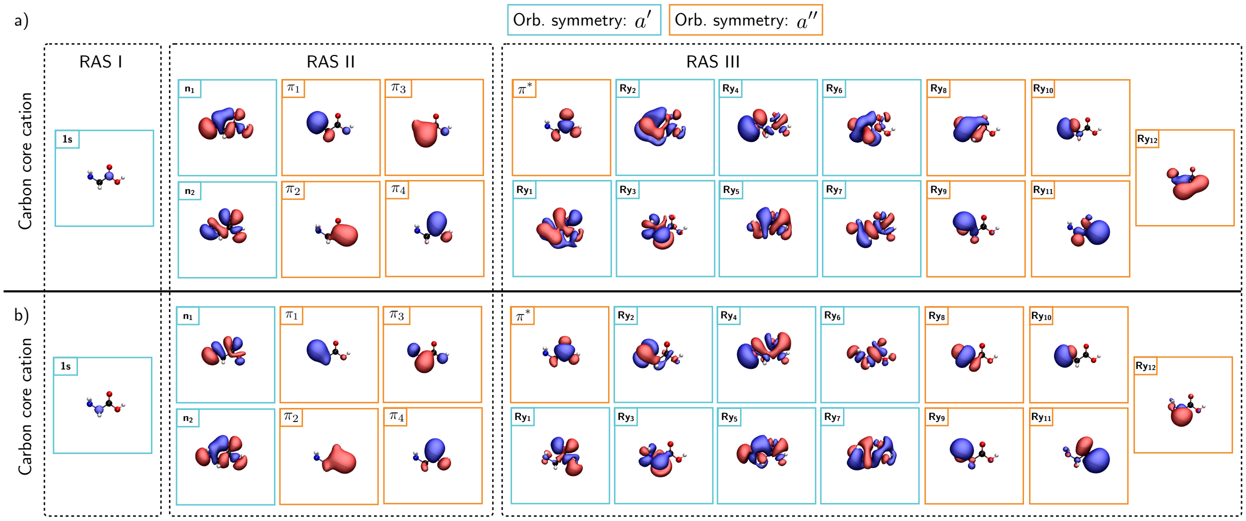

Figure S19: Active space used to compute O K-edge XPS of glycine in the "linear" conformation. a) Optimized AS used to compute the O_H XPS signal. b) Optimized AS used to compute the O_C XPS signal.

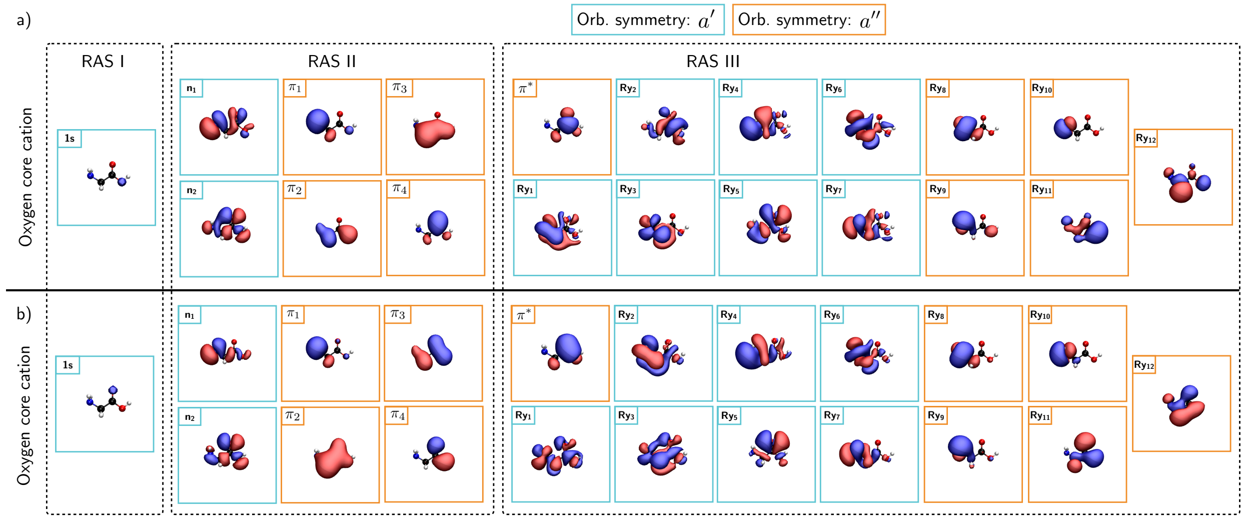

Figure S20: Active space used to compute C K-edge XPS of glycine (conformer A). a) Optimized AS used to compute the C_O XPS signal. b) Optimized AS used to compute the C_{H_2} XPS signal.

Table S21: Core excited states of a' symmetry that give rise to the nitrogen K-edge XANES of glycine (conformer A). The orbital involved in the excitation process are highlighted in figure S14-(a).

Root	ΔE	$\|TDM\|^2$	Configuration	Weight
1	401.37	4.34e-04	$1s^{[1]}Ry_3{}^{[1]}$	0.78
			$1s^{[1]}\pi_4{}^{[1]}Ry_3{}^{[1]}\pi^{*[1]}$	0.07
2	402.90	5.64e-05	$1s^{[1]}Ry_2{}^{[1]}$	0.11
			$1s^{[1]}Ry_5{}^{[1]}$	0.68
			$1s^{[1]}\pi_4{}^{[1]}Ry_5{}^{[1]}\pi^{*[1]}$	0.05
3	403.77	1.08e-04	$1s^{[1]}Ry_2{}^{[1]}$	0.68
			$1s^{[1]}Ry_5{}^{[1]}$	0.11
			$1s^{[1]}\pi_4{}^{[1]}Ry_2{}^{[1]}\pi^{*[1]}$	0.05

Table S22: Core excited states of a'' symmetry that give rise to the nitrogen K-edge XANES of glycine (conformer A). The orbital involved in the excitation process are highlighted in figure S14-(b).

Root	ΔE	$\lvert TDM\rvert^2$	Configuration	Weight
2	402.84	1.48e-03	$1s^{[1]}\pi^{*[1]}$	0.12
			$1s^{[1]}Ry_5{}^{[1]}$	0.67
			$1s^{[1]}\pi_4{}^{[1]}\pi^{*[1]}Ry_5{}^{[1]}$	0.06
3	405.22	7.41e-04	$1s^{[1]}Ry_8{}^{[1]}$	0.76

Table S23: Core excited states of a' symmetry that give rise to the oxygen K-edge XANES of glycine (conformer A). The orbital involved in the excitation process are highlighted in figure S15-(a).

Root	ΔE	$\lvert TDM\rvert^2$	Configuration	Weight
1	536.24	7.71e-04	$1s^{[1]}Ry_6{}^{[1]}$	0.63
			$1s^{[1]}\pi_4{}^{[1]}Ry_6{}^{[1]}\pi^{*[1]}$	0.08
			$1s^{[1]}\pi_4{}^{[1]}Ry_6{}^{[1]}\pi^{*[1]}$	0.21
2	538.21	1.60e-04	$1s^{[1]}Ry_2{}^{[1]}$	0.25
			$1s^{[1]}Ry_3{}^{[1]}$	0.38
			$1s^{[1]}\pi_4{}^{[1]}Ry_2{}^{[1]}\pi^{*[1]}$	0.09
			$1s^{[1]}\pi_4{}^{[1]}Ry_3{}^{[1]}\pi^{*[1]}$	0.11
3	539.13	2.21e-04	$1s^{[1]}Ry_2{}^{[1]}$	0.36
			$1s^{[1]}Ry_3{}^{[1]}$	0.27
			$1s^{[1]}\pi_4{}^{[1]}Ry_2{}^{[1]}\pi^{*[1]}$	0.12
			$1s^{[1]}\pi_4{}^{[1]}Ry_3{}^{[1]}\pi^{*[1]}$	0.08

Table S24: Core excited states of a'' symmetry that give rise to the oxygen K-edge XANES of glycine (conformer A). The orbital involved in the excitation process are highlighted in figure S15-(b).

Root	ΔE	$\lvert TDM\rvert^2$	Configuration	Weight
1	535.97	6.37e-04	$1s^{[1]}\pi^{*[1]}$	0.85
2	539.45	2.23e-04	$1s^{[1]}Ry_8{}^{[1]}$	0.77
			$1s^{[1]}\pi_4{}^{[1]}\pi^{*[1]}Ry_8{}^{[1]}$	0.09

Table S25: Core excited states of a' symmetry that give rise to the oxygen K-edge XANES of glycine (conformer A). The orbital involved in the excitation process are highlighted in figure S16-(a).

Root	ΔE	$\|TDM\|^2$	Configuration	Weight
1	535.37	6.27e-05	$1s^{[1]}Ry_3{}^{[1]}$	0.51
			$1s^{[1]}Ry_5{}^{[1]}$	0.29
2	536.35	1.68e-04	$1s^{[1]}Ry_3{}^{[1]}$	0.29
			$1s^{[1]}Ry_5{}^{[1]}$	0.52
3	537.38	3.46e-05	$1s^{[1]}Ry_3{}^{[1]}$	0.06
			$1s^{[1]}Ry_6{}^{[1]}$	0.76
4	539.35	1.20e-05	$1s^{[1]}Ry_2{}^{[1]}$	0.80

Table S26: Core excited states of a'' symmetry that give rise to the oxygen K-edge XANES of glycine (conformer A). The orbital involved in the excitation process are highlighted in figure S16-(b).

Root	ΔE	$\|TDM\|^2$	Configuration	Weight
1	532.94	2.46e-03	$1s^{[1]}\pi^{*[1]}$	0.90
2	537.44	1.28e-04	$1s^{[1]}Ry_8{}^{[1]}$	0.07
			$1s^{[1]}Ry_1 1^{[1]}$	0.79
5	539.64	9.11e-05	$1s^{[1]}Ry_8{}^{[1]}$	0.73
			$1s^{[1]}Ry_1 1^{[1]}$	0.07

Table S27: Core excited states of a' symmetry that give rise to the nitrogen K-edge XANES of glycine (conformer B). The orbital involved in the excitation process are highlighted in figure S17-(a). In red are labeled the transition originating from the intramolecular hydrogen bonding that are marked in figure 4 of the main manuscript with an asterisk.

Root	ΔE	$\|TDM\|^2$	Configuration	Weight
1	401.43	3.99e-04	$1s^{[1]}Ry_6^{[1]}$	0.75
			$1s^{[1]}\pi_4^{[1]}Ry_6^{[1]}\pi^{*[1]}$	0.08
2	403.77	1.94e-04	$1s^{[1]}Ry_3^{[1]}$	0.20
			$1s^{[1]}Ry_4^{[1]}$	0.49
			$1s^{[1]}\pi_4^{[1]}Ry_4^{[1]}\pi^{*[1]}$	0.06
3	404.55	7.11e-04	$1s^{[1]}Ry_3^{[1]}$	0.52
			$1s^{[1]}Ry_4^{[1]}$	0.21
			$1s^{[1]}\pi_4^{[1]}Ry_3^{[1]}\pi^{*[1]}$	0.05
4	405.72	3.77e-03	$1s^{[1]}Ry_4^{[1]}$	0.05
			$1s^{[1]}Ry_7^{[1]}$	0.71
			$1s^{[1]}\pi_4^{[1]}Ry_7^{[1]}\pi^{*[1]}$	0.07

Table S28: Core excited states of a'' symmetry that give rise to the nitrogen K-edge XANES of glycine (conformer B). The orbital involved in the excitation process are highlighted in figure S17-(b).

Root	ΔE	$\|TDM\|^2$	Configuration	Weight
1	402.91	1.24e-03	$1s^{[1]}Ry_3^{[1]}$	0.44
			$1s^{[1]}Ry_4^{[1]}$	0.36
2	403.96	3.86e-04	$1s^{[1]}Ry_3^{[1]}$	0.34
			$1s^{[1]}Ry_4^{[1]}$	0.45
3	406.42	7.78e-04	$1s^{[1]}Ry_5^{[1]}$	0.76

S4.7 Formaldehyde

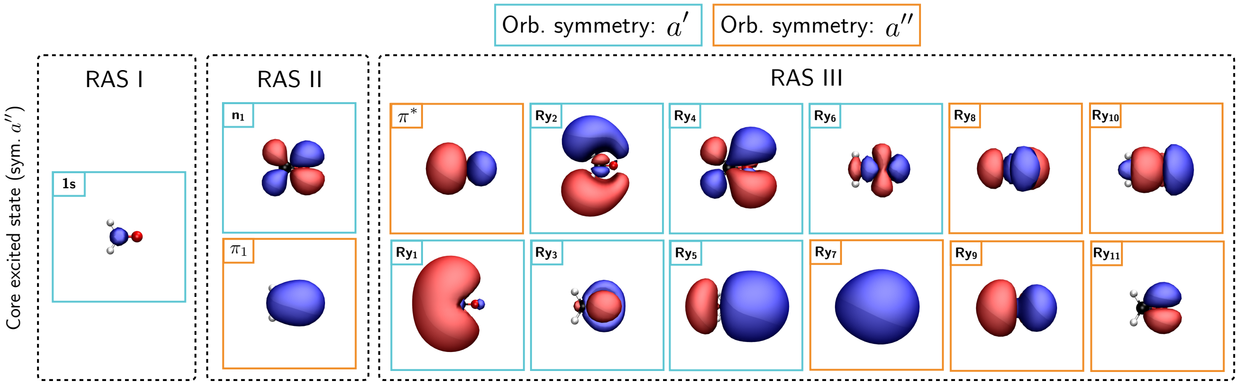

Figure S21: Active space used to compute C K-edge XANES of formaldehyde trough the DHO model.

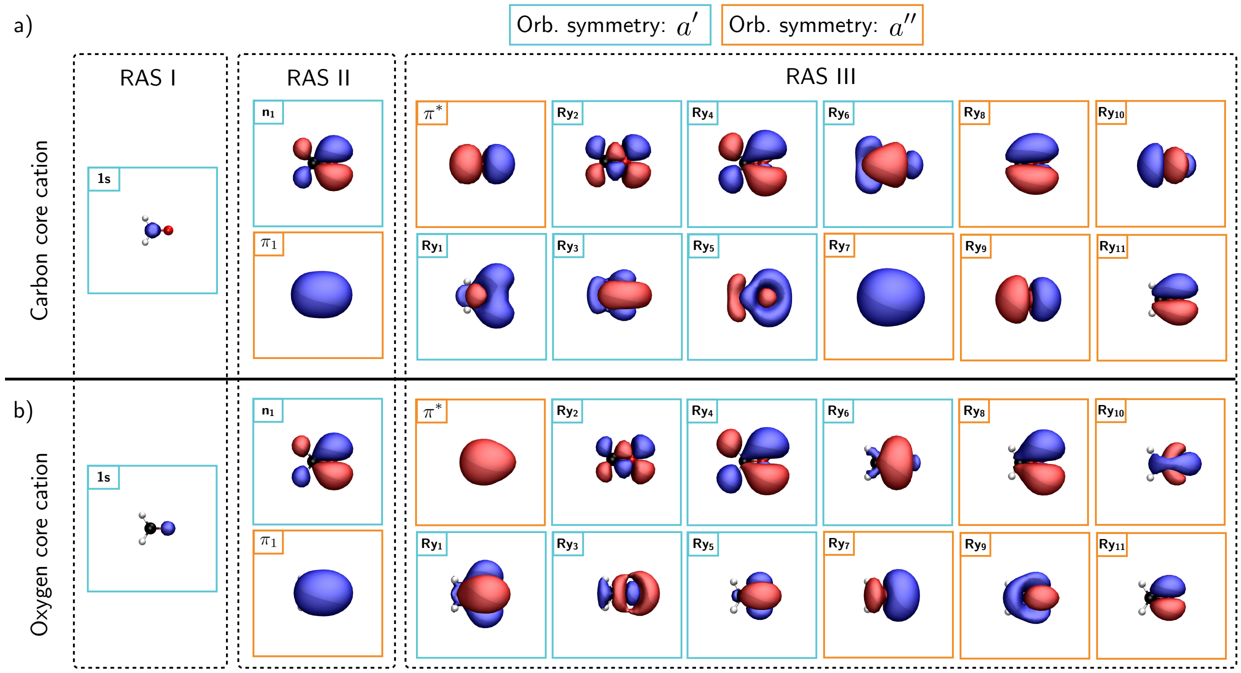

Figure S22: Active space used to compute C and O ionization potentials of formaldehyde. a) Optimized active space used to compute C IP. b) Optimized active space used to compute O IP.

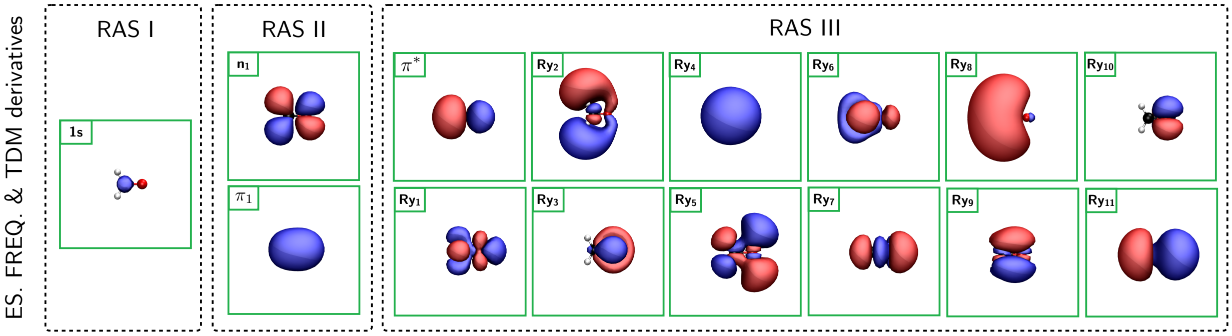

Figure S23: Active spaces of formaldehyde used to compute; a) Frequencies and normal modes of the $1s^{[1]}\pi^{*[1]}$ state in its minimum; b) transition dipole moments derivatives in Cartesian coordinates. Note that, in contrast with the other cases, no symmetry is employed in these computations.

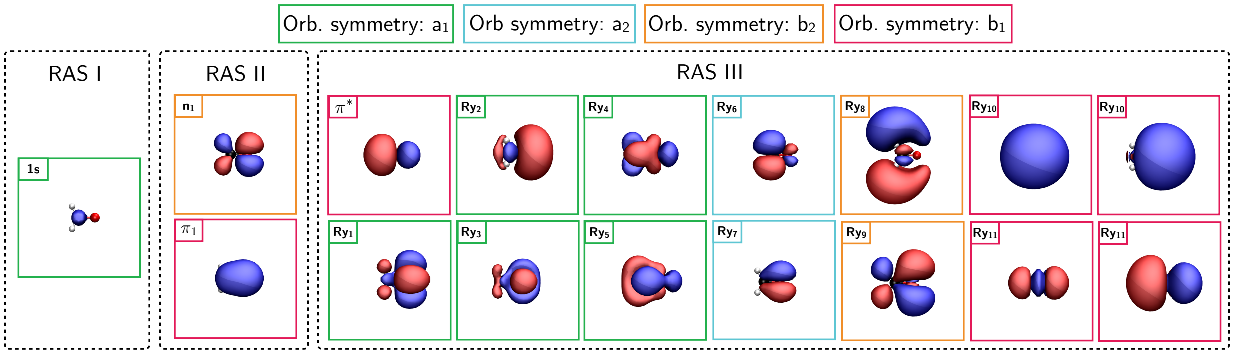

Figure S24: Active space of formaldehyde used to optimize the molecular structure of the $1s^{[1]}\pi^{*[1]}$ state. Note that, in contrast with the other cases, this computation employ the C_{2v} symmetry.

S5 Normal modes

In the following we report some information about the normal modes of formaldehyde and Vinyl fluoride, as these have been explicitly referred to in the main text.

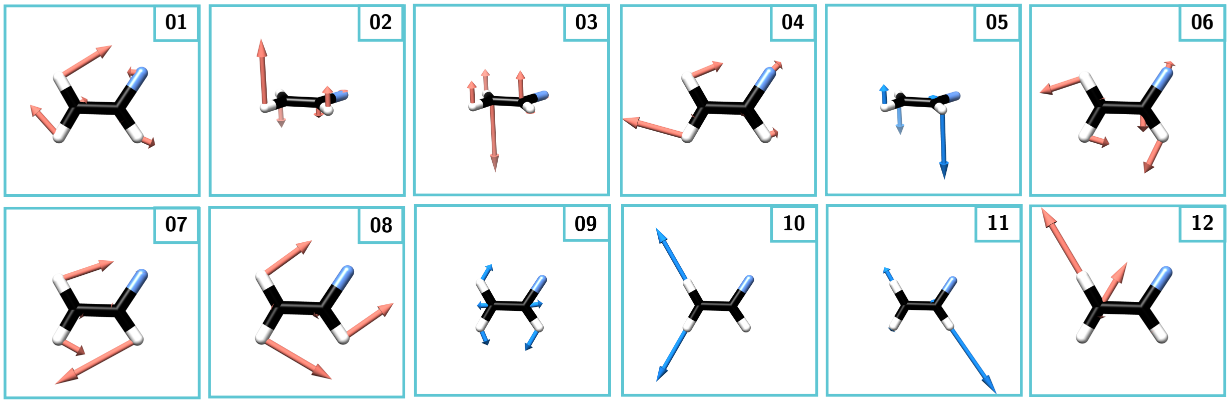

Figure S25: Graphical representation of Vinyl fluoride normal modes. Red and blue arrows here are used to point out respectively, positive and negative displacements along each single normal mode.

Table S29: Normal mode and frequency of Vinyl fluoride. A graphical representation of the normal modes is given in figure S25

Modes	Red. mass (amu)	Freq. (cm^{-1})
01	2.35	485
02	1.494	735
03	1.39	869
04	2.27	937
05	1.10	965
06	2.86	1189
07	1.20	1357
08	1.18	1434
09	4.08	1709
10	1.06	3209
11	1.10	3247
12	1.12	3318

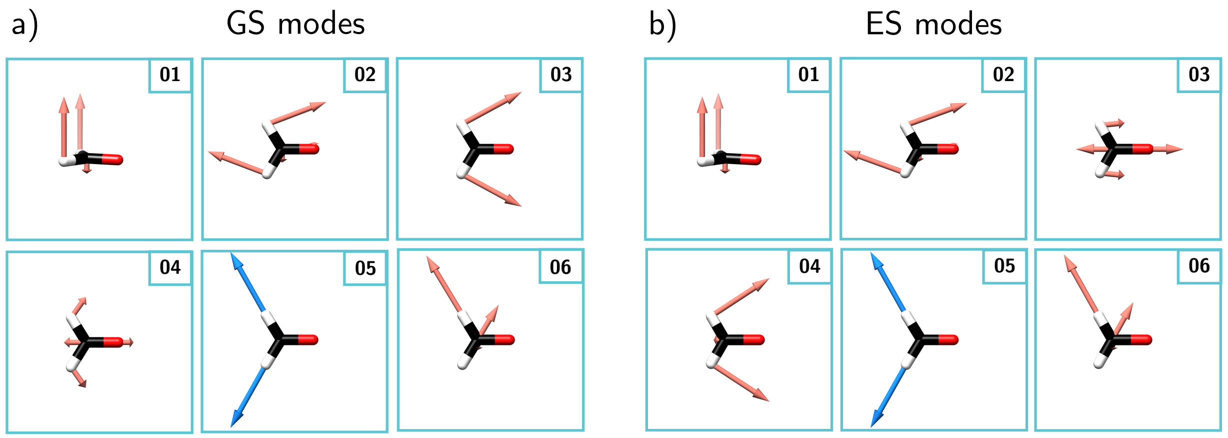

Figure S26: Graphical representation of formaldehyde normal modes for the ground state (a) and for the $1s^{[1]}\pi^{*[1]}$ state (b). Red and blue arrows here are used to point out respectively, positive and negative displacements along each single normal mode.

Table S30: Normal mode and frequency of Formaldehyde computed on the top of the GS minimum. A graphical representation of these normal modes is given in figure S26-(a).

Modes	Red. mass (amu)	Freq. (cm^{-1})
01	1.36	1195
02	1.33	1278
03	1.17	1547
04	5.50	1753
05	1.04	2970
06	1.12	3042

Table S31: Normal mode and frequency of Formaldehyde computed on the top of the $1s^{[1]}\pi^{*[1]}$ state minimum. A graphical representation of these normal modes is given in figure S26-(b).

Modes	Red. mass (amu)	Freq. (cm^{-1})
01	1.32	223
02	1.32	1255
03	5.86	1383
04	1.15	1637
05	1.04	3541
06	1.13	3710

S6 Shake-up transitions

Shake-up transitions are mainly described by two-electron excitations configurations. These involve the excitation of a core electron combined with a valence transition. Shake-up states are of interest because: (a) being two-electron excitations, only multi-configurational methods can be used to target them; (b) These states are dark in common XANES setups (being dominated by double-electron configurations), but can nonetheless become bright in the case of TR-XANES, as core-level transitions from a valence excited state; (c) They could be used to probe the valence electronic structure trough X-ray stimulated Raman processes.

The following table reports the shake-up features observed for the series of ethylenes/fluoro substitued ethylenes. We compare, for these systems, the difference between the GS $\rightarrow$ $1s^{[1]}\pi^{*[1]}$ transition and the GS $\rightarrow$ $1s^{[1]}\pi^{[1]}\pi^{*[2]}$ transition (shake-up feature) with the $\pi\pi^*$ excitation energy. In doing so, we are guided by the idea that the shake-up transition could be interpreted as the results of two subsequent excitations: first, the core-excitation is performed, and, on the top of that, a valence excitation follows. The shake-up energy is then viewed as the sum of the core-excited state energy (for example 1s$\rightarrow$ π^*) and the valence excitation energy (for example $\pi \rightarrow \pi^*$). Interestingly, we observe that the simple picture proposed above is approximate, as energy blue- and red-shifts are observed when the shake-up state is obtained through different core-excitations.

Table S32: Outline of the chemical shift of the shake-up features for the fluorosubstituted ethenes/ethylenes compared with $\pi\pi^*$ excitation energies of these systems.

Molecule	Excited core	$E_{1s^{[1]}\pi^{[1]}\pi^{*[2]}-1s^{[1]}\pi^{*[1]}}$ (eV)	$E_{\pi^{[1]}\pi^{*[1]}}$ (eV)
ethylene	C_{H_2}	8.2	8.3
Vinyl fluoride	C_{HF}	7.4	8.2
	C_{H_2}	9.0	8.2
1,1-difluoroethene	C_{F_2}	6.8	8.1
	C_{H_2}	10.0	8.1
cis-1,2-difluoroethene	C_{HF}	8.5	8.1
tetrafuoroethene	C_{F_2}	7,9	8.5

S7 XANES of fluorinated ethanes

The sensitivity of the XPS and XANES techniques to the local/chemical environment, is clearly demonstrated by studying the carbon K-edge of a set of fluorinated ethylenes. The substitution of an hydrogen with a strongly electronegative fluorine, alters the binding energy of the nearby carbon, thereby increasing its effective nuclear potential experienced by the electrons. This translates into a blue shift of the carbon core-excited state energies in the substituted systems with respect to the bare ethylene.

If both carbons are equally functionalized (cis-1,2-difluoroethylene and tetrafluoroethylene of Figure S27(d) and S27(e), respectively), they are chemically indistinguishable and the carbon core orbitals degeneracy is retained. The XANES spectra, in these cases, show a blue shift of the 1s $\rightarrow \pi^*$ transition which increases with the degree of fluorination. In particular, the main XANES peak experiences a blue-shift from ~285 eV (C_{H_2}) through ~287.5 eV (C_{HF}) to ~291 eV (C_{F_2}). If, in contrast, the two carbons have a different fluorination degree (Figure S27(a) and S27(b)), both chemical equivalence and core orbitals degeneracy are lost. These systems (vinyl fluoride and 1,1-difluoroethylene of Figure S27(b) and S27(a), respectively) show a splitting of the 1s $\rightarrow \pi^*$ transition. In particular, while the fluorinated carbon exhibits a strong blue-shift of the 1s $\rightarrow \pi^*$ transition, the unmodified carbon exhibits only a minor 0.5 eV blue-shift from its original position, a second-order effect due to the altered chemical environment of its first neighbor.

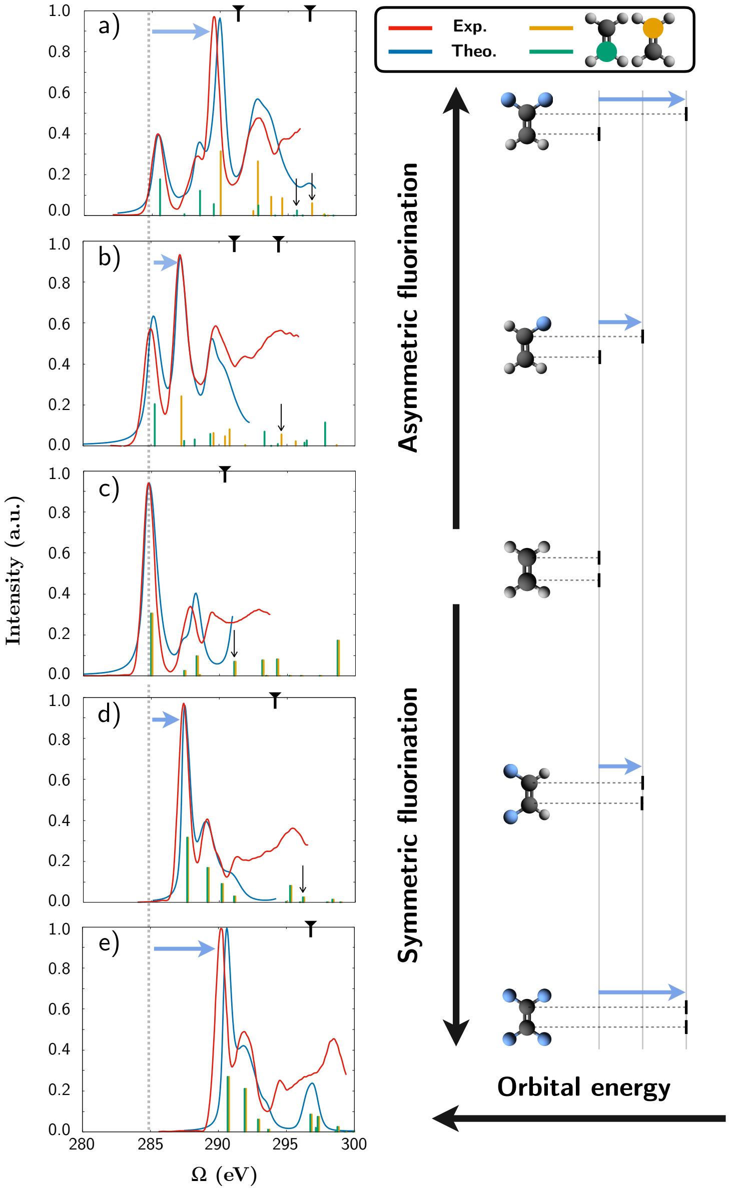

Figure S27: *Left*: XANES spectra for some fluorinated ethanes/ethylenes at the carbon K-edge. The contributions of individual core-transitions are highlighted with sticks of different color (green and orange, respectively), while the total spectra are depicted in blue. The red curve represents the experimental spectra from Ref. 3. The theoretical spectra are displayed with their line-shape below the experimental IP threshold (indicated by the black mark on the top edge of the spectra), whereas above it they are reported only as sticks. Shake-up (or mixed core-valence) states have been highlighted with black arrows. No shift has been applied to the theoretical spectra. The blue arrows highlight the fluorination-induced blue-shift. *Right*: schematic representation of the dependence of the molecular orbital (and in particular of the carbon core orbital) energies on the degree of fluorination.

S8 Glycine: single-configuration vs multi-configurations

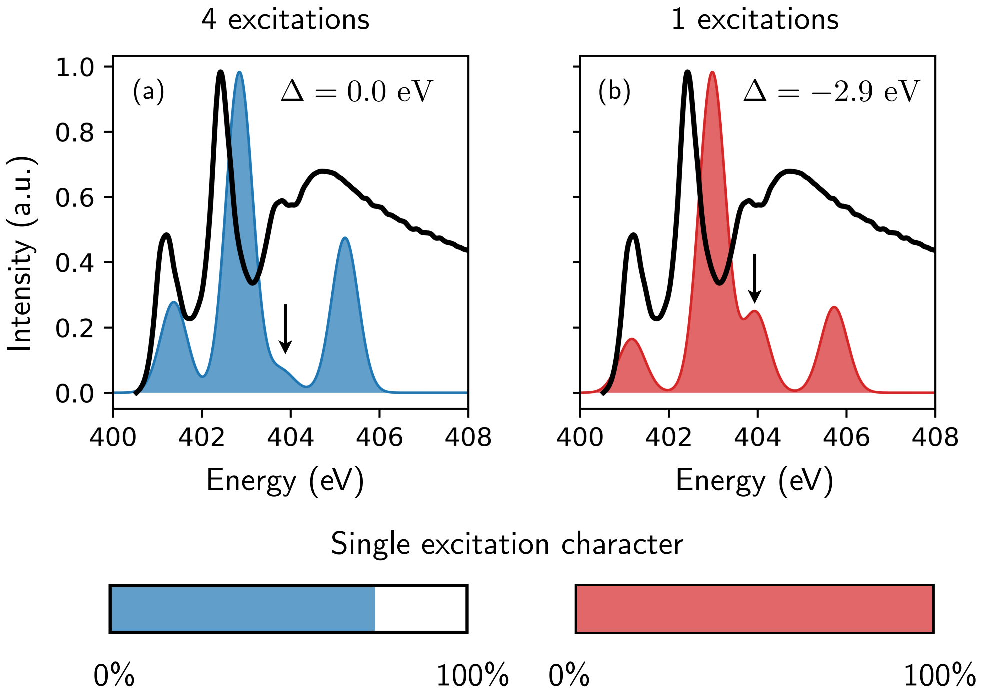

Figure S28: Comparison of the glycine most stable conformer spectra obtained employing (a) multiple-excitations as described in the computational details above, and (b) restricting the configuration state functions space to a single electron excitation. The transition discussed in the main text which shows a strong intensity dependence when single- and multiple-excitations are considered, is highlighted by a black arrow. A shift of -2.9 eV was applied to the spectrum in (b) to match the energy position of the higher level of theory in (a) and facilitate the comparison. The percentage of single-excitation character is reported, for both calculations, at the bottom of the Figure.

S9 The C−F example

In this section we provide additional details about the C−F example reported in the main text, by showing how the DHO model can be used to signal a significant change in the excited state geometry, and therefore a potential issue for the applicability of the DHO model itself.

Let us compare the ionization of the different centers of Vinyl fluoride, reported in Table S33 in terms of mode dependent Huang-Rhys (HR) factors and reorganization energies (λ). One notices that HR and λ are higher when the fluorine is ionized, compared to the core-ionization of C_{HF} or C_{H_2}. Common HR factors in optical spectroscopy are rarely as large as 0.5, while the fluorine core ionization leads to HR factors much greater than one. In these cases, the DHO model would predict an extremely large vibronic coupling, accompanied by a significant change in the excited state equilibrium geometry with respect to the GS minimum geometry. Indeed we note that such large values may signal an issue in the applicability of the DHO itself: as an example, we report that the optimization of the F 1s core cation ground state (performed at the same RASPT2 level of the core cation calculations reported above), reveals that the core ionization process leads to the dissociation of C−F bond.

In passing, we note that the sign of the mode-dependent displacement that connects the (neutral) ground state minimum to the core cation minimum (also reported in Table S33), can give valuable information about the ionization induced structural changes. In fact, the displacement along the C−F stretching (mode 4, also depicted in Figure S25) is positive at the fluorine K-edge and negative at the carbon K-edge, signaling an elongation and a contraction of C−F bond distance, respectively.

Table S33: DHO parameters for the ionization of the different centers of Vinyl fluoride.

K-edge	Mode	Freq. (cm^{-1})	Displ. (Bohr$\times\sqrt{(amu)}$)	HR (n.a.)	λ (eV)
C_{HF}	04	937	-0.18	0.13	0.02
	07	1357	0.16	0.14	0.02
	11	3247	0.06	0.51	0.02
C_{H_2}	04	937	-0.48	0.91	0.11
	06	1189	-0.27	0.37	0.05
	10	3209	0.10	0.15	0.06
F	04	937	0.61	1.43	0.17
	06	1189	0.73	2.62	0.39
	09	1708	0.32	0.74	0.16

In following the same steps of the C−F example reported in the main text, we evaluated the same DHO parameters also for core-excitations, initially focusing on the 1s→ π^* transition. It is noted that the total reorganization energy always increases: from ca. 0.7 to 1.14 eV for F, from 0.06 to 0.2 eV for C_{HF} and from 0.2 to 0.3 eV for C_{H_2}.

Table S34: DHO parameters for the 1s→ π^* transition of the different centers of Vinyl fluoride.

K-edge	Mode	Freq. (cm^{-1})	Displ. (Bohr$\times\sqrt{(amu)}$)	HR (n.a.)	λ (eV)
C_{HF}	06	1189	0.24	0.28	0.04
	09	1709	-0.24	0.40	0.08
	11	3247	0.12	0.21	0.08
C_{H_2}	09	1709	-0.24	0.42	0.09
	10	3209	0.18	0.45	0.18
F	04	937	1.00	3.88	0.45
	06	1189	0.97	4.65	0.69

Finally, we compare the DHO parameters for different types of fluorine core-excited states: in particular, we focus on the excitation of a 1s electron to either Ry, π^* or σ^* orbitals, which present an increasing anti-bonding character. As discussed in the main text, we observe here that the population of a Ry virtual orbital produces an almost neutral effect on the distortion of the excited state geometry (the HR values, in fact, are comparable or smaller to the ones obtained for the fluorine core-ionization); at variance, the population of π^* and σ^* induces a significant increase in the HR factors, and therefore a large change in

the molecular structure of the excited state. In particular, the population of a σ^* orbital leads to HR factors of about 11, that are clearly too large to be realistic.

Table S35: DHO parameters for different fluorine core excite states of vinyl fluoride.

Excitation	Mode	Freq. (cm^{-1})	Displ. (Bohr$\times\sqrt{(amu)}$)	HR (n.a.)	λ (eV)
1s$\rightarrow$ Ry	04	937	0.51	1.02	0.12
	06	1189	0.56	1.57	0.23
	08	1434	0.31	0.57	0.10
1s$\rightarrow$ π^*	04	937	1.00	3.88	0.45
	06	1189	0.97	4.65	0.69
1s$\rightarrow$ σ^*	04	937	1.69	11.1	1.29
	06	1189	1.55	11.8	1.75

The DHO is therefore proven to be a feasible model for the carbon K-edge, where HR values remain relatively small. Still, the presence of HR values close to one may constitute a warning that some modes can have different frequency in the GS and in the ES. Interestingly, in the case of F K-edge, the DHO itself signals its inability of correctly describing some transitions, displaying extremely large HR values that can be used as an indication of extreme phenomena such as the bond dissociation (which is indeed observed in the case of F ionization).

S10 Duschinsky and Herzberg-Teller

In the following we outline some technical details about the Duschinsky and HT effects that give rise to the vibrational resolved spectra of formaldehyde presented in Figure 7-(b,c) of the main text. In particular: in Table S36 we show the frequency of the GS and $1s^{[1]}\pi^{*[1]}$ state; in Table S37 we show the structure of the transformation matrix M [4] that projects the $1s^{[1]}\pi^{*[1]}$ state normal modes onto the basis of the GS normal modes; finally, in Table S38 we show the transition dipole moment derivatives along the normal coordinates of $1s^{[1]}\pi^{*[1]}$.

Table S36: Normal coordinates shift and frequency change computed for formaldehyde within the Duschinsky rotation framework. The "Schift" column represents the shift vector $\boldsymbol{K}$. [4]

Mode	Shift (Bohr$\times\sqrt{(\text{amu})}$)	Freq. GS (cm^{-1})	Freq. $1s^{[1]}\pi^{*[1]}$ (cm^{-1})
1	0.32e-03	1195	223
2	-0.96e-03	1278	1255
3	-0.61e+01	1547	1383
4	0.15e+02	1753	1637
5	0.86e+01	2970	3541
6	0.16e-01	3042	3710

Table S37: Transformation matrix M from the normal mode of $1s^{[1]}\pi^{*[1]}$ state to those of the GS for formaldehyde. The largest values are highlighted in red. Note the strong mixing between mode 3 and mode 4.

Modes	1	2	3	4	5	6
1	0.99945	-0.00000	0.00004	0.00024	0.00003	0.00001
2	0.00000	0.99866	0.00029	-0.00033	0.00001	0.02785
3	-0.00021	0.00010	0.59269	0.80533	-0.01229	-0.00005
4	-0.00011	0.00044	-0.80543	0.59267	-0.00609	-0.00018
5	-0.00004	0.00011	0.00238	0.01351	0.99990	-0.00418
6	-0.00001	-0.02831	-0.00011	0.00021	0.00418	0.99954

Table S38: Transition dipole moments derivatives along the normal coordinates of $1s^{[1]}\pi^{*[1]}$ state of formaldehyde.

Modes	x	y	z
1	-0.0000052	0.0000001	-0.0000075
2	0.0000004	0.0000000	-0.0003846
3	-0.0000004	0.0000000	0.0001210
4	-0.0000002	0.0000000	0.0007860
5	-0.0000001	0.0000000	0.0004649
6	0.0000011	0.0000000	-0.0010520

References

(1) Abramavicius, D.; Palmieri, B.; Voronine, D. V.; Šanda, F.; Mukamel, S. Coherent Multidimensional Optical Spectroscopy of Excitons in Molecular Aggregates: Quasiparticle versus Supermolecule Perspectives. *Chemical Reviews* **2009**, *109*, 2350–2408.

(2) Segatta, F.; Nenov, A.; Nascimento, D. R.; Govind, N.; Mukamel, S.; Garavelli, M. iSPECTRON : A simulation interface for linear and nonlinear spectra with ab-initio quantum chemistry software. *Journal of Computational Chemistry* **2021**,

(3) McLaren, R.; Clark, S. A. C.; Ishii, I.; Hitchcock, A. P. Absolute oscillator strengths fromK-shell electron-energy-loss spectra of the fluoroethenes and 1, 3-perfluorobutadiene. *Physical Review A* **1987**, *36*, 1683–1701.

(4) Bloino, J.; Biczysko, M.; Santoro, F.; Barone, V. General Approach to Compute Vibrationally Resolved One-Photon Electronic Spectra. *Journal of Chemical Theory and Computation* **2010**, *6*, 1256–1274.
